# Embracing Co-Design and Interprofessional Teamwork to Build an Innovative Dashboard for a National Social Needs Screening and Referral Clinical Intervention in the Veterans Health Administration: Design and Development Study

**DOI:** 10.2196/81846

**Published:** 2026-04-13

**Authors:** Lauren E Russell, Sarah M Leder, Jaime Boris, Alicia J Cohen, Christopher W Halladay, Kathleen M Mitchell, Sydney C Ruggles, Andrea Berkheimer, Chava Sonnier, Michelle C Wilcox, Ernest Moy, Meaghan A Kennedy

**Affiliations:** 1 Office of Health Equity, Veterans Health Administration, United States Department of Veterans Affairs, 810 Vermont Ave NW, Washington, DC, 20420, United States, 1 2028094182; 2 Center of Innovation in Transformative Health Systems Research to Improve Veteran Equity and Independence, Veterans Health Administration, United States Department of Veterans Affairs, Providence, RI, United States; 3 Department of Family Medicine, Alpert Medical School, Brown University, Providence, RI, United States; 4 Department of Health Services, Policy, and Practice, School of Public Health, Brown University, Providence, RI, United States; 5 New England Geriatric Research, Education, and Clinical Center, VA Bedford Healthcare System, United States Department of Veterans Affairs, Bedford, MA, United States; 6 Social Work Services, James E. Van Zandt Veterans Affairs Medical Center, United States Department of Veterans Affairs, Altoona, PA, United States; 7 Mental Health Services, George E. Wahlen Veterans Affairs Medical Center, United States Department of Veterans Affairs, Salt Lake City, UT, United States; 8 Department of Family Medicine, Chobanian and Avedisian School of Medicine, Boston University, Boston, MA, United States

**Keywords:** data dashboard, social needs, social determinants, social determinants of health, veterans

## Abstract

**Background:**

Assessing Circumstances and Offering Resources for Needs (ACORN) is a US Department of Veterans Affairs (VA) clinical intervention designed to identify and address social needs to improve health and well-being among all veterans. We co-designed the ACORN Dashboard to facilitate access to real-time social needs and intervention data for VA clinical care teams and leadership.

**Objective:**

This study aimed to (1) describe the iterative development of the ACORN Dashboard, (2) assess end user feedback and Dashboard usage, and (3) discuss the role of social needs dashboards in facilitating continuous quality improvement in health care settings.

**Methods:**

An interprofessional team of subject-matter experts and end user feedback contributed to the design. Phase 1 included more than 7 months of weekly working meetings. We initially constructed a wireframe in Microsoft PowerPoint, then translated it into a prototype in Power BI, a data visualization software. Using Microsoft Power BI, we built data visualizations to communicate population-level sociodemographic and ACORN screening data. Through feedback sessions, staff from 8 VA medical centers (VAMCs) reviewed the prototype and recommended improvements regarding the Dashboard’s purpose, content, and usability. Phase 2 involved 6 weeks of weekly working meetings, where we developed and iteratively refined 5 written drafts of clinically relevant variables for potential inclusion in the Patient-Level Data Page. This list informed a Power BI prototype. We also developed the ACORN Implementation Map page in Power BI to display implementation locations and settings. We again used feedback sessions with 8 VAMCs to review and refine the newly added pages and discuss improvements. To assess usage, we obtained metadata from a VA-specific Power BI report and user experience data from an ACORN VAMC survey.

**Results:**

The ACORN Dashboard displays national data that are updated daily, reflecting 83,546 screens administered across 82 VAMCs facilities between July 1, 2021, and April 30, 2025. The Dashboard was viewed 18,192 times by 2251 unique users, and, on average, 263 (SD 91.2) unique users viewed the Dashboard every month between October 1, 2023, and April 30, 2025. Dashboard variables include the number of screens completed, sociodemographic characteristics of veterans screened, prevalence of social needs, and interventions provided to address needs. Phase 1 semistructured feedback sessions included recommendations for a page with patient-level data to supplement the population-level pages, incorporation of additional filters to select specific data, and development of a user guide. In phase 2, key insights included enhancement of end users’ ability to search by veteran or staff name, guidance about screening frequency, changing the display order of variables, and the inclusion of variable definitions.

**Conclusions:**

Using co-design to develop, maintain, and continually refine data dashboards enhances implementation of social screening and interventions in health care settings. In addition to supporting individual-level patient care, population-level dashboard data inform continuous quality improvement, promote health equity, and identify gaps in services to address identified needs.

## Introduction

As electronic health record (EHR) usage has increased over the past two decades, so too has the volume of patient medical and demographic data collected and maintained by health systems [1]. Data-driven technologies in health care settings include tools such as clinical and quality dashboards [2,3] that function through the collection, analysis, and application of individual- and population-level health information [4]. The integration of data-driven technologies into health systems and the use of these technologies as part of routine clinical care can enhance quality and patient safety, improve health outcomes, inform tailored clinical care and the provision of social and medical services, and contribute to public health and health services research [4-6]. However, data-driven tools should be developed and implemented with a thoughtful and intentional approach, with the end users at the center of the design.

Coinciding with the rise in technology use in health care, the value of medical and social care integration has been increasingly recognized among health care professionals, policymakers, and funders [7-11]. This has led health systems to improve their efforts to collect information on health-related social needs (herein referred to as “social needs”), which are social and economic aspects of individuals’ lives that affect their health and well-being [12]. Examples of these needs include housing instability, food insecurity, unemployment, lack of access to telehealth, and social isolation. Although many health systems now routinely collect information on social needs, which is an accreditation requirement of the Joint Commission [11], social needs data are typically not readily available to health professionals in a user-friendly way (ie, without requiring a comprehensive chart review) to inform individual-level care and population health management [13,14].

The US Department of Veterans Affairs (VA) has often been at the forefront of integrating social and medical care through its provision of health care and social services supplemented by its financial investment in innovation and quality improvement (QI) efforts. In 2012, the VA implemented universal screening for housing instability [15], followed in 2017 by universal screening for food insecurity [16]. In addition to these efforts, the VA also offers a range of resources and supportive services for veterans with unmet social needs, such as supportive housing services, vocational rehabilitation, assistance for justice-involved veterans, digital device access and digital health literacy support, and peer-support services [17-21]. Given its robust infrastructure of internal supports and collaborations with Veteran Service Organizations, the VA is uniquely positioned to respond to the medical and social needs of the patients it serves. Developed in 2018, Assessing Circumstances and Offering Resources for Needs (ACORN) is a VA clinical intervention aimed at identifying and addressing social needs to improve health and well-being among all veterans [22,23]. Since 2021, ACORN has been implemented in partnership with the VA Office of Health Equity and the VA National Social Work Program, Care Management and Social Work Services. By the end of April 2025, clinical care teams had implemented ACORN in a variety of clinical settings in 82 VA medical centers (VAMCs) across 37 states and Puerto Rico. Adoption has largely been supported by implementation tools and technical support provided through a national ACORN Community of Practice [24]. Since the ACORN pilot in 2019 to 2020 [22], frontline clinical teams using ACORN have consistently shown interest in reviewing their own data. Doing so allows them to identify the most frequently endorsed social needs within their VAMC or clinical setting, as well as the most common resources or referrals provided to veterans who report any social needs. During initial pilot phases, our team responded to data requests on a case-by-case basis, often responding to requests received via email or through secure instant message (ie, Microsoft Teams). As screening expanded to more VAMCs, it became apparent that fulfilling ad hoc, VAMC-specific data requests was an unsustainable method of technical support. Therefore, we focused on developing a data sharing method that would enable VA leadership and VAMCs to access their data directly. To facilitate access to real-time social needs screening and intervention data for VA clinical care teams and leadership, we iteratively developed and refined the ACORN Dashboard, incorporating end user feedback at multiple time points. Similar to the approach used to develop the ACORN screening tool in 2018 [22], we actively involved end users in the ACORN Dashboard design process.

Data dashboards providing clinical care teams with immediate access to patient information can enhance adherence to quality guidelines and improve patient health outcomes [25]. Although existing literature describes the development of clinical dashboards [26-29], little has been published to date describing the development, usage, and effectiveness of social needs dashboards intended for use in health care settings. In this paper, we aimed to (1) describe the iterative development of the ACORN Dashboard, (2) examine usage and feedback about the ACORN Dashboard, and (3) discuss the role of data dashboards in facilitating continuous QI in health care settings.

## Methods

### Project Overview

**Figure 1.**
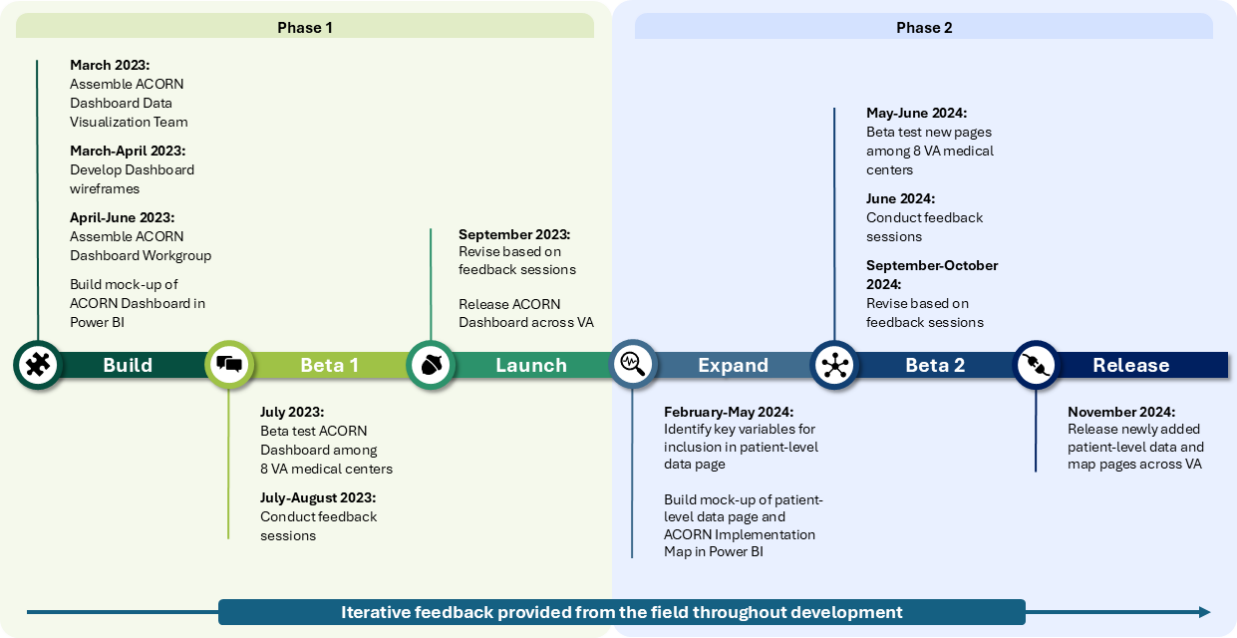
Assessing Circumstances and Offering Resources for Needs (ACORN) Dashboard development timeline for phases 1 and 2 (March 2023 to November 2024). This timeline depicts how each phase of development consisted of 3 key steps: in phase 1, build, beta test, and launch; and in phase 2, expand, beta, and release. The ACORN Dashboard data visualization team, which developed the initial wireframes of the Dashboard in PowerPoint, consisted of 8 individuals with diverse backgrounds and expertise, including clinical social work, nursing, public health, clinical informatics and data analysis, and visual design. A smaller team of members from the ACORN Dashboard data visualization team then formed the ACORN Dashboard Workgroup. This workgroup transformed the PowerPoint wireframes into a functional prototype in Power BI, a data visualization software. This same Workgroup supported the beta testing in both phase 1 and phase 2, as well as the expansion of the Dashboard in phase 2. The arrow at the bottom of the figure depicts the incorporation of iterative feedback throughout development. VA: US Department of Veterans Affairs.

The ACORN Dashboard was developed over two phases: (1) initial development, consisting of co-design and build of population-level data pages; and (2) expansion, which included the addition of a Patient-Level Data Page based on end user feedback (Figure 1). The ACORN Dashboard Workgroup consisted of 4 key members: a public health practitioner involved in co-developing ACORN and co-leading VA-wide implementation (LER); a biostatistician with robust knowledge of VA data, including ACORN-specific data capture elements, and coding expertise (CWH); a data analyst with a background in social work and expertise in the ACORN dataset and Microsoft Power BI (SML); and an exercise physiologist with a background in health administration and experience in building Power BI reports (JB).

Co-design principles were incorporated throughout to ensure that the product was aligned with end user preferences and the needs of interprofessional clinical staff and leaders. Through feedback sessions in both phases, 37 staff (including 4 staff across 3 VAMCs who participated in both phase 1 and phase 2) from a total of 13 VAMCs reviewed the design and provided critical insights regarding the usability of, and content included in, the Dashboard.

### Ethical Considerations

As this work was conducted for the purposes of program evaluation and QI, it was determined by the VA Central Institutional Review Board to be a nonresearch activity and did not require regulatory review. As a nonresearch activity, formal informed consent was not obtained; staff who participated in pilot testing of the ACORN Dashboard and corresponding feedback sessions were informed that their participation was voluntary, that notes would be taken, and that findings would be used to inform the development and enhancement of end user tools. Staff participated within their routine roles and did not receive specific compensation for engaging in pilot testing or feedback sessions.

### ACORN Dashboard Development

#### Phase 1: ACORN Dashboard Creation

##### Phase 1: Co-Design and Initial Build

We embraced a co-design approach, incorporating interprofessional subject-matter expertise and end user input from the outset. To ensure a functional “end product,” we formed the ACORN Dashboard data visualization team in March 2023. We recruited VA staff knowledgeable in the importance of population and individual-level data to ensure the value of the Dashboard to both frontline clinical staff and VA leaders, whom we anticipated would be our end users. Additionally, we included data analysts, specifically, subject-matter experts who possessed a comprehensive understanding of the ACORN dataset and those who would be able to create a functional end product. This interprofessional team also possessed the knowledge and expertise to determine which data were clinically relevant and feasible to display in a dashboard. The final data visualization team consisted of 8 individuals with expertise spanning clinical social work, nursing, public health, clinical informatics and data analysis, and visual design.

The data visualization team met weekly through the beginning of April 2023. This team brainstormed the purpose, content, and layout of the ACORN Dashboard and created several wireframes (ie, mockups of dashboard pages including tables, charts, and figures). To ensure all members of this team could access and make edits on the wireframes, we used Microsoft PowerPoint slides to create mockups of each page that we would ultimately create in Power BI, the data visualization software used across the Veterans Health Administration. Using Power BI Desktop enabled us to design, build, and iteratively refine the Dashboard, while uploading it to Power BI Service (or Power BI Server) generated a shareable link that could be disseminated to all VA employees. Although we initially planned for the Dashboard to be a single page, it quickly became evident that we would need to expand to multiple pages and determine which data could be grouped together, for example, a quick snapshot of the most frequently requested ACORN data (ie, number and percentage of those endorsing each social need), veterans’ sociodemographic information, and the most commonly provided resources and referrals to address needs. Once the data visualization team reached consensus on a complete wireframe, we gathered feedback from staff in the VA Office of Health Equity and members of the ACORN Leadership Team, which consists of Office of Health Equity and National Social Work Program leadership and staff, health services researchers who are also VA primary care physicians, data scientists or analysts, and biostatisticians.

Incorporating this feedback, a subset of members from the data visualization team then formed the smaller ACORN Dashboard Workgroup (hereafter the “workgroup”) to code and build the initial Dashboard. The workgroup met weekly between mid-April 2023 and the end of June 2023 to discuss progress made on the Power BI draft of the Dashboard, review outstanding issues, and verify next steps. As the primary goal of the Dashboard was to provide data collected across the entire VA, including all 139 VAMCs, we quickly established a threshold to protect patient privacy. Adhering to the VA Office of Health Equity standard practice for protecting protected health information (PHI), if any cell in any table had fewer than 13 patients, the true count would be hidden.

After approximately 10 weeks, we completed the first draft of the Dashboard and uploaded it to the Office of Health Equity’s Power BI online workspace.

##### Phase 1: Beta Test Dashboard

In early July 2023, we recruited staff from 8 VAMCs to serve as “beta testers.” Medical centers were recruited through ACORN Community of Practice calls and direct email outreach. We included medical centers in various stages of ACORN implementation (ie, preimplementation, implementation, and sustainment) and across different clinical settings and geographic locations to ensure a diverse range of perspectives and feedback on the initial dashboard design and content.

##### Phase 1: Collect End User Feedback Through Semistructured Feedback Sessions

Staff from each of the 8 VAMCs agreed to test the Dashboard over the course of a month (July 2023) and participate in a 1-hour semistructured feedback session with 2 to 3 workgroup members between late July and early August 2023. Prior to conducting semistructured feedback sessions, we sent a list of usability questions to each VAMC (Supplemental File 1 in Multimedia Appendix 1). We then used these questions to guide our feedback sessions. During each session, 1 member of the workgroup took detailed notes in a structured template to capture quotes, barriers to and facilitators of Dashboard usage, and opportunities for improvement, while the other members asked questions.

##### Phase 1: Analyze End User Feedback From Semistructured Feedback Sessions

After the conclusion of the 9 feedback sessions (1 VAMC requested 2 feedback sessions to accommodate varying staff schedules), findings were summarized in a template based on the feedback session guide, and discrepancies in the interpretation of any of the responses were resolved through consensus discussion between the workgroup members in attendance at the respective feedback session. Additionally, the workgroup members who participated in the feedback sessions reviewed responses for each template domain to identify the emergence of key themes. The full workgroup then reconvened to review the summary template, which included the compilation of “action items” from all the feedback sessions. First, we discussed the key insights identified by workgroup members who participated in the feedback sessions. We reviewed the summary template section by section, both for action item clarity and to resolve any concerns about an action item’s categorization (eg, if one team member thought a piece of feedback in the “layout” section should be captured in the “content” section). Second, we wrote a “general recommendation” response for each action item and grouped them into three categories: (1) action items with a clear plan for resolution, (2) action items needing additional research or follow-up from our team to glean additional insight or assess the feasibility, and (3) action items outside of the scope of the Dashboard. Third, we assessed the feasibility of addressing the key action items. Feasibility was assessed based on two criteria: (1) whether our team possessed the skill set and expertise to make the change prior to the planned release on September 30, 2023; and (2) whether we had the ability to make the modification within the constraints of Power BI when applicable for action items. Finally, the action items deemed feasible were prioritized based on anticipated time to complete them, with those taking the least amount of time to resolve being at the top of the list. We addressed all action items on the priority list before the announcement and online release of the Dashboard to all VA staff at the end of September 2023.

### Phase 2: Dashboard Expansion

#### Phase 2: Refinement and Expansion of Dashboard Features

The impetus for phase 2 was 2-fold. First, multiple end users noted the importance of accessing patient-level data during phase 1 feedback sessions (see *Results* section). Furthermore, after the initial ACORN Dashboard launch (phase 1), we received multiple requests from end users to incorporate a Patient-Level Data Page that could be used to inform clinical practice and support care teams in following up with individual patients. The requests often included specific patient-level data elements that end users sought to use to appropriately tailor clinical care. The workgroup used these requests to develop an initial list of variables for inclusion in a Patient-Level Data Page. We also reviewed 2 existing VA dashboards that include patient-level data—the Food Insecurity Summary Screen Dashboard developed by the VA Food Security Office and the Primary Care Equity Dashboard [30] developed by researchers at the VA Center for Health Equity and Research Promotion. To protect PHI on the Patient-Level Data Page, we used a dynamic filtering feature on Power BI Service and VA data tables that limits internal users’ data access permissions based on their assigned VAMC (location) and the overall data permissions granted by the VA.

Between the end of February 2024 and the beginning of April 2024, we developed 5 drafts of key variables for inclusion. Once members of the workgroup reached consensus on the complete list of patient-level variables, we reviewed and gathered feedback from staff in the Office of Health Equity and members of the ACORN Leadership Team. In addition to the Patient-Level Data Page, we also agreed to develop an ACORN Implementation Map to display VAMCs and clinical settings in which ACORN had been implemented thus far.

#### Phase 2: Beta Test Dashboard

After completing a draft version of the Patient-Level Data Page and the ACORN Implementation Map in Power BI, we shared a link to our test version in mid-May 2024 with 8 VAMCs. As phase 2 beta testing included the Patient-Level Data Page, which relies on Veteran PHI, we only included sites that had already implemented ACORN in this testing. This ensured that participants could view real, local patient (specifically PHI) data. Staff at each VAMC could only see patient-level data that they would otherwise have clinical access to view within the VA EHR. Similar to phase 1, we recruited staff at these VAMCs to serve as “beta testers” through the ACORN Community of Practice and direct email outreach based on their involvement in ACORN. The 8 VAMCs agreed to test the Patient-Level Data Page over the course of a month (mid-May to mid-June 2024) and to meet for a 45‐ to 60-minute feedback session with 2 to 3 members of the Workgroup between mid- to late-June 2024.

#### Phase 2: Collect End User Feedback Through Semistructured Feedback Sessions

Similar to phase 1, we conducted semistructured feedback sessions with each beta testing VAMC. We aimed to obtain insights to refine the Patient-Level Data Page and the ACORN Implementation Map to make sure they were (1) useful to VAMCs, (2) being interpreted as intended, and (3) appropriately tailored to meet the needs of frontline staff. During each session, 1 member of the workgroup served as the lead or facilitator, while the other served as the primary notetaker or recorder. However, both the lead or facilitator and primary notetaker or recorder took detailed notes in a structured template to capture responses about features that should be added or removed and feedback on the content, layout, and usability of the Patient-Level Data Page and any requested changes to the ACORN Implementation Map.

#### Phase 2: Analyze End User Feedback From Semistructured Feedback Sessions

After completing all 8 feedback sessions, 2 members of the workgroup summarized the findings in a template based on the feedback session guide and resolved discrepancies in the interpretation of any of the findings through consensus discussion. These workgroup members also reviewed responses from each VAMC to identify key themes gleaned from feedback sessions.

The full workgroup reconvened in September 2024 to review the summary template and a list of key action items put together by the workgroup members who facilitated the feedback sessions. We reviewed the key action items one by one and placed them into the following categories: (1) feasible changes to be made to existing content; (2) feasible variables and features to incorporate before release; (3) deletions to existing content; and (4) follow-up and outstanding questions or issues, which included notes about requested changes that were not feasible due to Power BI limitations or the available time window to make modifications. Between September and October 2024, we revised the Patient-Level Page to address the findings in categories 1 to 3 and worked to both answer remaining issues in category 4 and communicate to the “beta testers” which of the requested changes were not feasible.

### Dashboard User Data

#### Usage

We used the VA-developed “Usage Metrics” Power BI report, which allows Power BI workspace administrators to access metadata related to the reports they have created. The Usage Metrics report allowed us to track the total number of Dashboard views and the number of unique viewers over time.

#### User Experience

As part of an annual survey used to understand how VAMCs are implementing ACORN, staff were asked to complete 2 questions about how frequently they used the ACORN Dashboard and how useful they found it. Staff from all 63 VAMCs that had implemented ACORN between October 2023 and September 2024 were invited to complete the survey via a Qualtrics link distributed via email. Invited staff were asked to provide 1 response per VAMC. When we received more than 1 response for a given VAMC, the most complete response (ie, most questions completed) was used for analyses.

## Results

### Phase 1 End User Feedback Session Findings

Between late July and early August 2023, 9 feedback sessions were conducted with the 8 VAMCs who served as “beta testers” of the ACORN Dashboard. A total of 27 staff participated, with a median of 3 (IQR 1‐8) staff per VAMC. Participants represented a broad range of professions, including clinical leadership, health informatics, and frontline staff (ie, physicians, social workers, and nurses).

Overall, respondents found the ACORN Dashboard informative, intuitive, easy to navigate, and user-friendly. However, staff suggested several content and layout changes and noted several points of confusion or difficulty (Textbox 1). They reported that a variety of staff roles or disciplines would likely use the ACORN Dashboard, including clinical care team members or frontline staff (eg, social workers and nurses), health informaticists, executive leadership teams (eg, VAMC directors and chiefs of staff), and team members of locally tailored efforts to improve health equity and address health disparities. Key refinements made based on phase 1 end user feedback included modifying the names of certain variables, domains, and categories on the Dashboard; adding additional filters to allow staff to further refine their data search by quarter within a fiscal year and by domain on the Domain Trends page; developing a user guide and frequently asked questions resource; and providing the data source for included variables. This end user feedback also prompted the Workgroup to begin conceptualizing a Patient-Level Data Page (phase 2).

Textbox 1. **Key insights and refinements from Assessing Circumstances and Offering Resources for Needs (ACORN) dashboard development phase 1 (July to August 2023).**

Include patient-level data

Modify language for ease of use (ie, change “–” to “<13”)

Enhance available filters (eg, fiscal year and quarter)

Develop a user guide and frequently asked questions resource

Clarify data source (ie, data derived from the ACORN electronic health record template)

The key insights and refinements presented in Textbox 1 are based on recommendations gleaned from 9 feedback sessions conducted with 27 staff across 8 VAMCs for phase 1 of the ACORN Dashboard development between July and August 2023. Participants represented clinical leadership, health informatics, and care team members or frontline staff (ie, physicians, social workers, and nurses).

### Phase 2: End User Feedback Session Findings

In June 2024, we conducted feedback sessions with staff from 8 VAMCs (n=3, 37.5%, of which also participated in phase 1 beta testing) who agreed to serve as “beta testers” of the Patient-Level Data Page and the ACORN Implementation Map. Overall, 13 staff representing a variety of roles or disciplines, including social work, mental health, and nursing, participated in the feedback sessions, with a median of 1.5 (IQR 1‐3) attendees per VAMC.

Feedback regarding the Patient-Level Data Page was overall positive, with one VAMC reporting, “This is perfect. It’s exactly what we’re looking for,” and another sharing, “We really wanted to know which patients were screened and things like that. We’ve really been hoping for something like this...this will be very helpful.” The majority of staff found the page easy to follow and understand. Several VAMCs mentioned their appreciation for the ability to export data from Power BI to Excel or PDF, noting that this functionality made it easier to share data with colleagues and VA leadership. Staff generally agreed that the most critical domains for inclusion were the active suicide flag indicator (an indicator for suicide risk in the VA EHR), positive social needs domains, and the Veterans’ Care Assessment Needs score (a VA-validated risk prediction model) [31]. One VAMC noted that they found the Patient-Level Data Page included too many domains, which made it difficult to easily navigate the page and duplicated information readily available in veterans’ EHR (eg, sex, race, ethnicity, and sexual orientation). This VAMC also noted that many of the domains were not crucial data points for informing follow-up care. Staff also proposed several enhancements (Textbox 2). Key refinements to the Patient-Level Data Page made based on phase 2 end user feedback included enhancing the end user’s ability to search by veteran or staff name; incorporating clarification about screening frequency (ie, date of next recommended screen); modifying the display order of the variables; providing data definitions for variables in an updated version of the user guide and frequently asked questions resource; and improving the overall readability and usability of the Dashboard contents.

Textbox 2. **Key insights and refinements from Assessing Circumstances and Offering Resources for Needs Dashboard development phase 2 (July 2024).**

Enhance end user’s ability to search by veteran or US Department of Veterans Affairs staff name

Incorporate guidance about screening frequency

Modify order of variables

Define included variables

Improve readability and usability (eg, increase font size and add alternative text)

The key insights and refinements presented in Textbox 2 are based on recommendations gathered during the 8 feedback sessions conducted with 13 staff across 8 VAMCs for phase 2 of the ACORN Dashboard development during July 2024. Participants represented clinical leadership and care team members or frontline staff from several disciplines (eg, social work, mental health, and nursing).

For the ACORN Implementation Map, staff were generally appreciative of this added feature, and no proposed changes were suggested during our feedback sessions.

### Final ACORN Dashboard Components (Includes Phase 1 and Phase 2)

**Figure 2.**
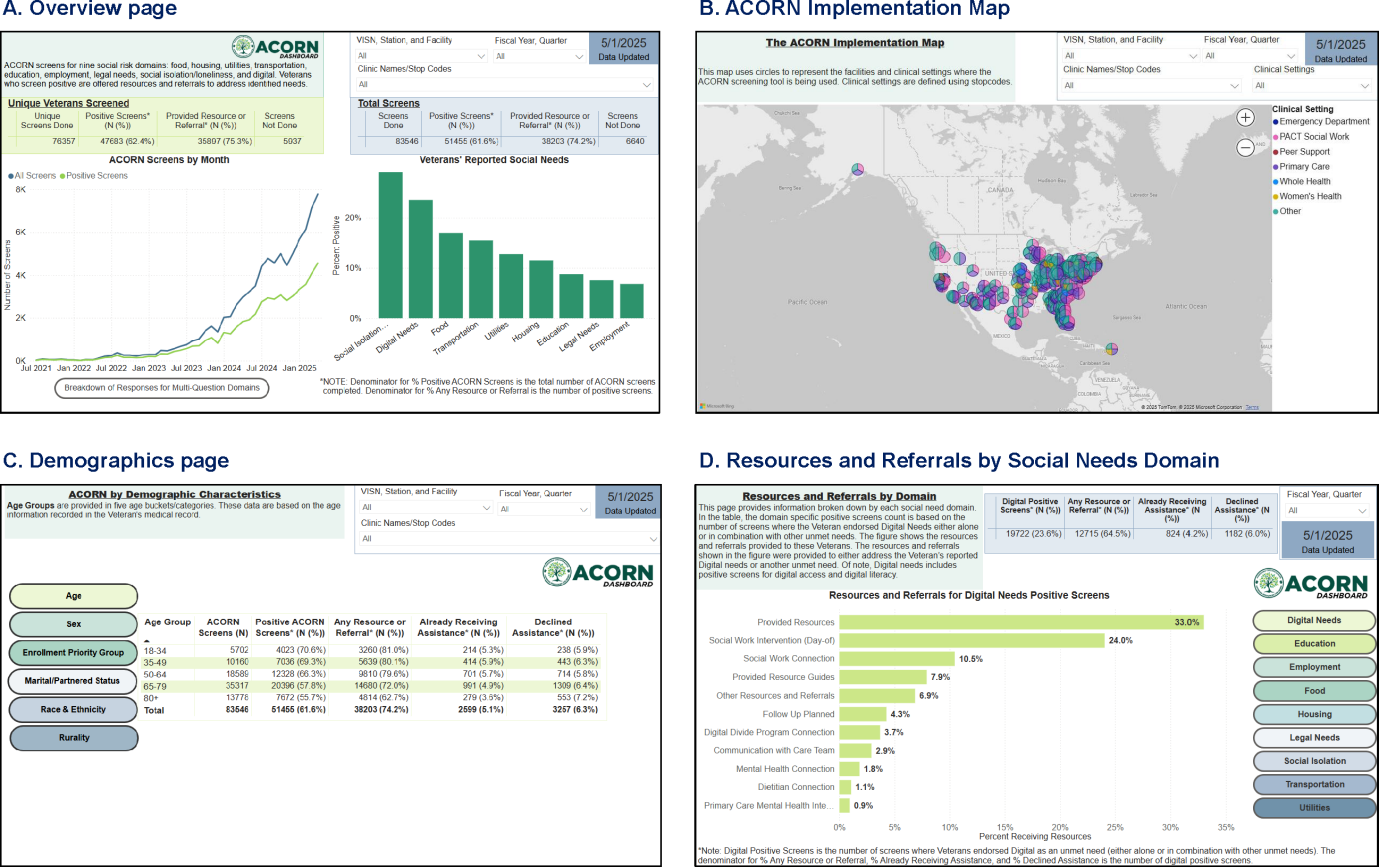
Assessing Circumstances and Offering Resources for Needs (ACORN) Dashboard page examples. (A) The Overview page; (**B**) the ACORN Implementation Map page; (**C**) the Demographics page; (**D**) the Resources and Referrals by Social Needs Domain page. As of May 1, 2025, the date on which the above screenshots were taken and included data gathered through April 30, 2025, the Dashboard reflected 83,546 screens among 76,357 unique patients across 82 Veterans Affairs Medical Centers since July 1, 2021. Among the 47,683 (62.4%) unique veterans screening positive for one or more social needs on ACORN, 35,897 (75.3%) received a resource and/or referral to a relevant VA or community service. Panels A, C, and D were developed as part of ACORN Dashboard development phase 1 (March to September 2023), while panel B was developed as part of ACORN Dashboard development phase 2 (February to November 2024). (The ACORN Dashboard continually updates daily, so the data shown are for the study period.)

The original ACORN Dashboard (phase 1) was launched in September 2023, and the Patient-Level Data Page and ACORN Implementation Map (phase 2) were released in November 2024 [24] (Figure 2). The overall aim of the ACORN Dashboard is to increase access to data collected through the ACORN screening tool in the VA EHR. The ACORN Dashboard helps VA care teams and leadership: (1) understand the social needs impacting patients, (2) track the types of resources and referrals provided to patients who screen positive on ACORN, and (3) observe how screening rates and the provision of resources and referrals vary by sociodemographic factors and over time. At the end of the study period, the Dashboard reflected 83,546 screens among 76,357 unique patients across 82 VAMCs since July 1, 2021. Among the 47,683 (62.4%) unique veterans screening positive for one or more social needs on ACORN, 35,897 (75.3%) received a resource and/or referral to a relevant VA or community service.

As previously noted, Dashboard variables, including screened veterans’ sociodemographic information, ACORN screening and resource and referral data, as well as clinical characteristics, are pulled from the VA Corporate Data Warehouse and are updated nightly at 11:59 PM Eastern Time (see Supplemental File 2 in Multimedia Appendix 2 for a complete list of variables).

### Dashboard Training and Tools

In conjunction with the initial release of the Dashboard (phase 1), members of the workgroup provided a tutorial for the ACORN Community of Practice (October 2023) and on a national VA webinar (January 2024). Additionally, email announcements were sent to internal VA staff and VAMCs involved in the ACORN Community of Practice, and an announcement was included on the ACORN SharePoint VAMC, an internal VA website available to all staff. Concurrent with the release of the Dashboard, the workgroup released the ACORN Dashboard User Guide and Frequently Asked Questions (FAQs; Supplemental File 2 in Multimedia Appendix 2). The User Guide and FAQs was created as an additional resource to provide information asynchronously and for those who might prefer a written reference. The guide contains a brief overview of the ACORN screening tool, a description of the rationale behind the Dashboard, and in-depth descriptions and screen captures of each Dashboard page. Crucially, the User Guide and FAQs provide explanations of features that could cause trouble for end users who are unfamiliar with Power BI. This includes information about the on-screen filters that allow users to limit their view by categories (eg, VAMC, clinic type, and fiscal year). In alignment with our co-design approach, the User Guide and FAQs were developed based on the review of summary notes and questions gathered from end user feedback sessions. After the Implementation Map page was created and Patient-Level Data Page was updated following the same principles as the Patient-Level Data Page (phase 2), a similar communications approach was taken. An overview of the new pages was provided to the ACORN Community of Practice (November 2024), and a more in-depth tutorial was provided to this group in April 2025. Additionally, descriptions and screenshots of the Patient-Level Data Page and ACORN Implementation Map, along with questions from the phase 2 end user sessions, were added to an updated User Guide and FAQs.

### ACORN Dashboard User Data

#### Usage

**Figure 3.**
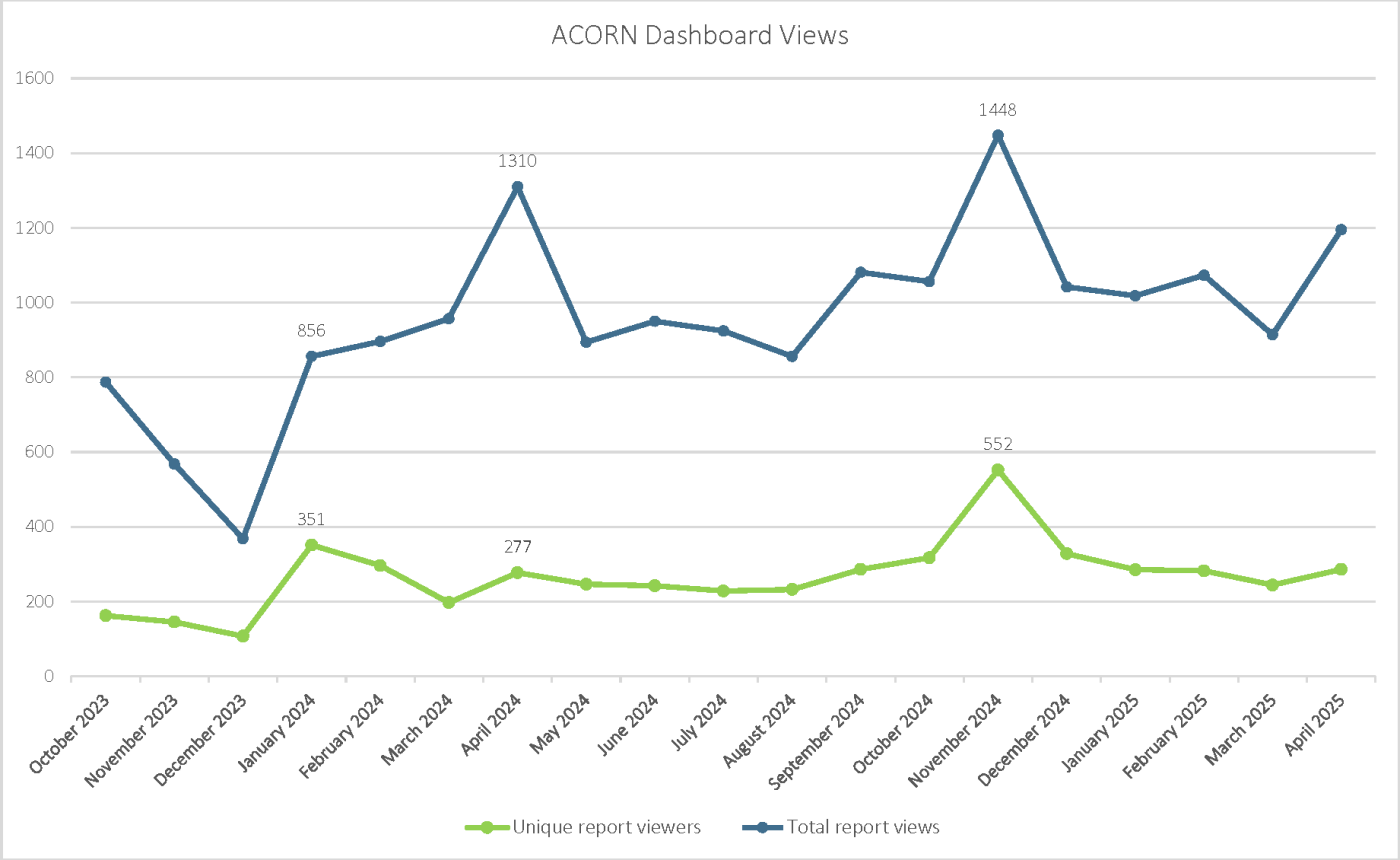
Assessing Circumstances and Offering Resources for Needs (ACORN) Dashboard unique and total views by month. October 2023 marks the first full month after the release of the ACORN Dashboard. Data from September 23, 2023, to September 30, 2023, are not shown. Between October 1, 2023, and April 30, 2025, the Dashboard was viewed 18,192 times by 2251 unique users. On average, 263 (SD 91.2) unique users viewed the Dashboard every month, with a range of 107 to 552.

Between October 1, 2023, and April 30, 2025, the Dashboard was viewed 18,192 times by 2251 unique users. On average, 263 (SD 91.2) unique users viewed the Dashboard every month, with a range of 107 to 552. Three notable spikes in the number of Dashboard views per month (Figure 3) correspond to key ACORN milestones. The first spike in January 2024 (856 total views and 351 unique views) corresponds to the release of the ACORN national template in the VA EHR, which was announced via an email from the VA Health Information Community of Practice and through the ACORN Community of Practice monthly calls, Microsoft Teams Channel chat, and ACORN SharePoint VAMC. The second spike in April 2024 (1310 total views and 277 unique views) coincides with the ACORN screening tool receiving endorsement from the VA Governance Board, which is composed of VA senior leaders who oversee operational activities to ensure decisions are prioritized and aligned with VA’s mission and the strategic direction set forth by the VA Under Secretary for Health. Many regional leaders and members of medical centers’ executive leadership teams reported receiving awareness of this change from their regional directors or others in their chain of command. Finally, the most recent spike in November 2024 (1448 total views and 552 unique views) corresponds to the release of the Patient-Level Data Page and ACORN Implementation Map, which was announced through the ACORN Community of Practice monthly calls, Teams Channel chat, and ACORN SharePoint VAMC.

#### User Experience

Of 63 invited VAMCs, 48 completed the annual VAMC survey in October 2024; of those, 48 completed a question on how frequently they used the Dashboard, and 46 completed a question on how useful they found it. More than half (54.1%) of the VAMCs reported “often” using the Dashboard, 20 (41.7%) reported “sometimes” using the Dashboard, and 2 (4.2%) reported “never” using the Dashboard. Nearly two-thirds (65.2%) of VAMCs reported that the Dashboard was “very useful,” 15 (32.6%) reported that the Dashboard was “somewhat useful,” and 1 (2.2%) reported that the Dashboard was “not useful at all.”

## Discussion

### Brief Summary of the ACORN Dashboard Co-Design Process and Usage

In this paper, we describe the iterative development, usage, and staff perceptions of a social needs dashboard. The overarching purpose of the ACORN Dashboard is two-fold. First, it is intended to help care team members, including clinicians, nurses, social workers, and other allied health professionals, review individual-level social needs, sociodemographic, and clinical data among veterans screened to guide additional outreach or follow-up and inform local QI efforts to ensure appropriate follow-up for veterans screening positive. Second, it provides population-level social needs data collected through the ACORN EHR template to care team members, local and regional leadership, and national leadership to understand the unmet social needs impacting veterans, identify gaps in the service delivery model, and inform future resource allocation in response to these needs. Using data gathered during this study period, the Dashboard displayed national data gathered from 82 VAMCs facilities during an approximately 4.5-year time span, with 83,546 ACORN screens administered among 76,357 unique veterans. Since the Dashboard’s launch, more than 2250 unique users viewed the Dashboard nearly 18,200 times. Phase 1 and phase 2 feedback sessions provided insight into end users’ goals and opportunities for Dashboard enhancements. Literature exists on the development of dashboards for patient quality and safety as well as those to address health disparities and promote health equity and to display social determinants (or drivers) of health in relation to certain disease states or clinical conditions [1,29,32-34]. However, to our knowledge, this is the first description of the development and use of a social needs dashboard intended to inform both individual-level clinical care and population-level health management.

### Alignment With Existing Literature

Using a co-design process, we leveraged the VA’s EHR to create a dashboard for a national VA social needs clinical intervention. Notably, this approach helped us to collaboratively create a data-driven tool that both meets end user needs and provides a range of staff, from clinical providers to senior VAMC and organizational leadership, with access to routinely collected social needs data that are updated daily [4,5,25]. This tool optimizes the use of clinical (individual-level) and population-level health information for end users, which ultimately promotes patient-centered care. Aligned with prior studies, our work demonstrates interest among health professionals in accessing clinical data collected from patients during the provision of routine clinical care [1,35]. The interprofessional co-design approach used to build the ACORN Dashboard also builds upon existing literature highlighting the importance of integrally involving health professionals throughout the development of patient data dashboards to enhance usage [36]. As prior research suggests [29,34], including health professionals, especially from a diverse range of backgrounds and disciplines, as we did in this work, can help ensure that included data are meaningful and clinically relevant to a broad audience.

The Dashboard also aligns with the principles of visual management systems used to monitor and improve care [37]. To maximize the likelihood of engagement and use of this tool, we used a co-design process to build and iteratively revise the ACORN Dashboard. Using this approach meant including relevant partners, the anticipated end users, from the outset. We sought to ensure that VA care team members and leaders relying on this real-time, point-of-care tool could seamlessly identify and use ACORN Dashboard visuals to communicate critical information [38]. As noted in other studies, including end users in the design phase is critical for electronic (or digital) health care tools whose effectiveness largely depends on high levels of care team member usage [39,40]. Additionally, the range of staff roles and disciplines of those involved in the data visualization team, Office of Health Equity, ACORN Leadership Team, and VAMC staff involved in feedback sessions provided valuable insights into diverse perspectives on use contexts and end user needs. This enhanced our understanding of what clinicians, care team members, and leaders perceived as most relevant for tailoring clinical care and informing population health management. Building a strong data visualization team with a diverse skill set played a crucial role in our ability to create an end product that was not only functional but also easy to navigate and aesthetically pleasing. Similar to other studies, engaging end users throughout the Dashboard development and refinement process likely contributed to the acceptability of the prototypes that were beta tested and discussed during phases 1 and 2 [39,41,42]. It may also have contributed to the consistent use of the Dashboard over time, as evidenced by user trends [30,43].

The Dashboard also aligns with the principles of visual management systems used to monitor and improve care [37]. To maximize the likelihood of engagement and use of this tool, we used a co-design process to build and iteratively revise the ACORN Dashboard. Using this approach meant including relevant partners, the anticipated end users, from the outset. We sought to ensure that VA care team members and leaders relying on this real-time, point-of-care tool could seamlessly identify and use ACORN Dashboard visuals to communicate critical information [38]. As noted in other studies, including end users in the design phase is critical for electronic (or digital) health care tools whose effectiveness largely depends on high levels of care team member usage [39,40]. Additionally, the range of staff roles and disciplines of those involved in the data visualization team, Office of Health Equity, ACORN Leadership Team, and VAMC staff involved in feedback sessions provided valuable insights into diverse perspectives on use contexts and end user needs. This enhanced our understanding of what clinicians, care team members, and leaders perceived as most relevant for tailoring clinical care and informing population health management. Building a strong data visualization team with a diverse skill set played a crucial role in our ability to create an end product that was not only functional but also easy to navigate and aesthetically pleasing. Similar to other studies, engaging end users throughout the Dashboard development and refinement process likely contributed to the acceptability of the prototypes that were beta tested and discussed during phases 1 and 2 [39,41,42]. It may also have contributed to the consistent use of the Dashboard over time, as evidenced by user trends [30,43].

### Application of the ACORN Dashboard and Facilitation of Continuous QI Efforts

The practice of creating and using data dashboards aligns with the VA’s efforts to not only be a high-reliability organization but also a high-equity-reliability organization [44]. High-equity-reliability organizations consider environmental factors that influence patient health and well-being and recognize that it is imperative to understand patients’ feelings and their values [44], which aligns with systematic efforts to identify and address social needs. Many VAMCs have sought to consider environmental factors through the incorporation of social needs screening and referral initiatives. Often, they have conducted this work through QI initiatives.

To inform continuous local and national QI efforts, we collect ongoing end user input to optimize clinical use. In addition, we provide 4 potential avenues for users to find technical assistance and other support [45]. The most accessible is the ACORN Dashboard User Guide and FAQs (described earlier). We also provide support through the ACORN Community of Practice and ACORN “Office Hours” monthly calls. These calls present opportunities for VAMCs to request and receive uniquely tailored technical assistance from members of the ACORN Leadership Team and learn about best practices from other VAMCs involved in the ACORN Community of Practice. Additionally, users can reach out to the Dashboard team using a designated VA email address to troubleshoot errors. To provide teams with the opportunity to access additional VAMC-specific data beyond the scope of what is provided through the Dashboard, our team developed an ACORN Data Pull Request Form in collaboration with colleagues in the Office of Health Equity. Through this process, we also offer consultations to support individual VAMCs in obtaining ACORN screening data for local QI and evaluation efforts. The focus of these requests usually includes in-depth data tailored to local priorities and a desire to track specific outcome measures. These processes keep multiple channels of communication open and have allowed for robust documentation of suggestions for consideration and improvements to the Dashboard.

Using the Dashboard to assess population-level data can support the identification of inequities and provide health systems with data needed to identify and fill gaps in services to address identified social needs. Similar to the VA Primary Care Equity Dashboard [30], we wanted to create a tool through which VA leaders could use the aggregate data to inform VAMC-level QI efforts and resource allocation, and care team members could “drill down” to clinic-level and patient-level data to tailor clinical interventions and enhance patient-centered care offerings. In alignment with existing guidance on incorporating equity into QI efforts [30,46,47], we recognized the importance of allowing staff to view stratified data. To this end, we built the Demographics page, which enables end users to view ACORN data stratified by age, sex, race and ethnicity, and other demographic characteristics.

### Insights and Implications for VA and Non-VA Health Settings

It is imperative to acknowledge that the VA is uniquely well positioned to screen for and address social needs, given its emphasis on interprofessional patient-aligned care teams, which include social workers embedded in primary care teams [48], and robust resources and supportive services to address the myriad social needs included in ACORN [17-21]. Although non-VA health systems may not have as many “in-house” comprehensive service offerings as the VA, the co-design process, including the timeline detailing the steps of phases 1 and 2, described in this paper could be used by other health systems to inform a similar process. Even if non-VA health systems are collecting social needs data through different screening instruments, the processes detailed herein, particularly the inclusion of interprofessional health professionals in the design and build and the approach to making iterative refinements made through both phases, could be applied to build a dashboard with individual- and/or population-level data. Additionally, the ACORN Dashboard User Guide & FAQs (Supplemental File 2 in Multimedia Appendix 2) can serve as a template for health systems (and potentially other organizations) looking to develop educational materials and resources for staff.

The link between social needs and health outcomes is well established; thus, collecting social needs data is now viewed by many as a standard of care [7,10,11,22,49-54]. First developed and used in a VAMC in New England [22], the ACORN clinical intervention has now been implemented in approximately 85 VAMCs in all 18 VA regions. In addition to the VA’s increased adoption of social needs clinical interventions in recent years, The Joint Commission established a National Patient Safety Goal standard in 2022 that requires hospitals to assess patients’ social needs and provide relevant connections to community resources and supportive services to address reported needs [11,54]. To meet this accreditation requirement, both VA and non-VA health systems must screen for, assess, and address social needs using tools such as ACORN [11]. We have received feedback from VAMCs that Dashboard data, including the data visualizations, has supported their efforts to meet this Joint Commission standard. Non-VA health systems may also be able to use a similar strategy if they develop data visualizations to display information gathered through social needs screening instruments used within their clinical settings and programs. Our team is conducting ongoing evaluation efforts to further explore and quantify this impact.

### Strengths and Limitations of the ACORN Dashboard

Notable strengths of the ACORN Dashboard include its daily updates, which provide near real-time data to staff and leadership. Using these data, care team members and frontline staff can optimize their provision of clinical care for veterans, track their metrics, and share their innovative work with veterans, other frontline staff, and local, regional, and national leadership. Its user-friendly design makes the ACORN Dashboard well suited to support program evaluation efforts at the local and national levels. The data captured in the Dashboard enable care teams to provide proactive and uniquely tailored services to veterans while optimizing resource use, which can result in more efficient and effective service delivery. Additionally, the development of the Dashboard minimizes delays in data access among care team members and frontline staff by reducing the need for the ACORN Leadership Team to respond to a large volume of VAMC-specific data requests manually. However, the Dashboard does have several limitations. The initial version (phase 1) was restricted to national-level, or aggregate, data; however, we resolved this limitation with the development of the “Patient-Level Data Page” (phase 2). Due to patient privacy concerns, the VAMC-level filters were not initially available for the “Co-Occurring Social Needs by Social Need Domain” and “Resources and Referrals by Social Need Domain” pages due to small cell sizes at the level of granularity for these domain-related data. However, the filters were added in June 2025 after nearly 100,000 screens had been completed across all VAMCs. The Dashboard also only includes data from VAMCs using the ACORN screening tool and the specific clinical settings in which they have implemented ACORN. As a result, data are not representative of all veterans enrolled in the VA, and data within certain social need domains may differ from other VA dashboards or published reports that include similar data (eg, food security).

### Limitations of the Approach Used to Build the ACORN Dashboard

Our findings should be interpreted within the context of several limitations. First, one beta testing VAMC in phase 1 was in preimplementation, meaning it had not yet started conducting ACORN screening, and therefore, staff were not able to view their own VAMC-level data during testing. However, their inclusion was intentional as we sought to solicit feedback from VAMCs at all phases of implementation. Those in preimplementation could provide valuable feedback regarding perceived usefulness and perspectives on how to use data from the Dashboard to increase buy-in among local leadership and frontline staff to support ACORN adoption. Additionally, findings may have been susceptible to selection bias because many of the VAMCs involved in our evaluation had a high level of buy-in and engagement. Although the intent of the Dashboard is to provide data to VA leaders at the national, regional, and VAMC levels, leadership was likely underrepresented in beta testing procedures (outside of national leadership from the VA Office of Health Equity and VA National Social Work Program). However, we solicited high-level feedback on the Dashboard components from members of the ACORN Partner Engagement Group, which comprised representatives from offices and programs across the VA and serves as an advisory body to inform the development of objectives and initiatives to support VA-wide implementation of ACORN [24]. Finally, although findings from the end user feedback sessions during phase 1 and phase 2 and the user data support acceptability and use of the ACORN Dashboard, a formal evaluation of ACORN Dashboard usage, satisfaction, and impacts across all VAMCs is needed.

### Conclusions

On the basis of continued uptake of the ACORN clinical intervention and use of the ACORN Dashboard, this paper adds critical information about engaging collaborators in a co-design process to build tools that inform patient care and promote the adoption of innovations within health systems. The development, maintenance, and iterative refinement of innovative data dashboards can support the implementation of social needs screening and referral interventions in health care settings. Documenting and tracking patient- and population-level data on social needs informs individual-level care and quality assurance in the provision of necessary follow-up, facilitates population health management, and helps identify health inequities and gaps in available services.

## Supplementary material

10.2196/81846 Multimedia Appendix 1 Assessing Circumstances and Offering Resources for Needs (ACORN) Dashboard development: phase 1 usability questions.

10.2196/81846 Multimedia Appendix 2 Assessing Circumstances and Offering Resources for Needs (ACORN) User Guide and Frequently Asked Questions.

## Abbreviations

ACORN: Assessing Circumstances and Offering Resources for Needs
EHR: electronic health record
FAQ: frequently asked question
PHI: protected health information
QI: quality improvement
VA: US Department of Veterans Affairs
VAMC: Veterans Affairs Medical Center
