# Supplementary material for: Embracing Co-Design and Interprofessional Teamwork to Build an Innovative Dashboard for a National Social Needs Screening and Referral Clinical Intervention in the Veterans Health Administration: Design and Development Study
Source: J Med Internet Res. 2026 Apr 13;28:e81846. doi: 10.2196/81846 (PMC13075635; doi:10.2196/81846)
Supplement: Multimedia Appendix 1 [file jmir-v28-e81846-s001.docx]

**Assessing Circumstances and Offering Resources for Needs (ACORN)**

**Dashboard Development, Phase 1**

**Beta/Pilot Tester – Dashboard Usability Questions**

1. What did you think of the dashboard in general?
   1. Content
   2. Layout
   3. Ease of use – how easy was it for you to navigate/find what you were looking for/understand?
2. What would you change about the dashboard?
   1. Content – anything you would add or remove?
   2. Layout – would you move/shift anything?
   3. Ease of use – what would make it easier to use?
3. We will walk through each page with your team and ask for specific comments/feedback.
   1. What information did you find most useful?
   2. Is there anything you found confusing or difficult to understand?
4. How do you anticipate using the dashboard?
   1. Who on the team uses or will likely use the dashboard the most – nurses, social workers, supervisors?
5. Any other comments or feedback you would like to share with us.
