# Supplementary material for: Embracing Co-Design and Interprofessional Teamwork to Build an Innovative Dashboard for a National Social Needs Screening and Referral Clinical Intervention in the Veterans Health Administration: Design and Development Study
Source: J Med Internet Res. 2026 Apr 13;28:e81846. doi: 10.2196/81846 (PMC13075635; doi:10.2196/81846)
Supplement: Multimedia Appendix 2 [file jmir-v28-e81846-s002.docx]

**
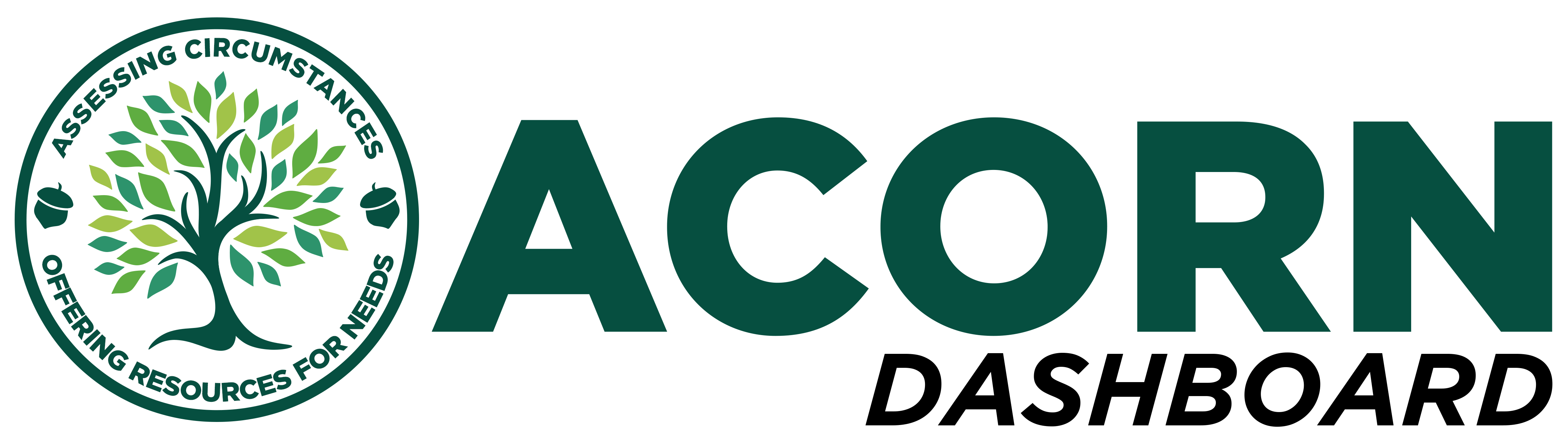
**

**Assessing Circumstances and Offering Resources for Needs (ACORN)**

**ACORN Dashboard**

**User Guide and FAQ**

*ACORN Dashboard User Guide & FAQ Developed by*

*Sarah M. Leder, MSW, Lauren E. Russell, MPH, MPP, Kathleen M. Mitchell, MPH, and Meaghan A. Kennedy, MD, MPH*

Contents

[ACORN Screening Tool 4](#_Toc193884168)

[Disposition Field / Action Steps in the ACORN CPRS Template 7](#_Toc193884172)

[ACORN Dashboard 8](#_Toc193884173)

[Purpose and Rationale 8](#_Toc193884174)

[A Note on Protected Health Information 9](#_Toc193884177)

[Data Definitions 10](#_Toc193884178)

[How to Use the ACORN Dashboard 12](#_Toc193884180)

[On-Screen Filters and Tooltip Feature 12](#_Toc193884181)

[Patient-Level Data Page Table Search 13](#_Toc193884182)

[ACORN Implementation Map 14](#_Toc193884183)

[Exporting Information from the Dashboard 14](#_Toc193884184)

[Pages on the ACORN Dashboard 15](#_Toc193884185)

[1. Before You Begin 15](#_Toc193884186)

[2. Overview Data 15](#_Toc193884187)

[3. Demographics 17](#_Toc193884188)

[4. Domain Trends 18](#_Toc193884189)

[5. Resources and Referrals 19](#_Toc193884190)

[6. Co-Occurring Needs by Domain 19](#_Toc193884191)

[7. Resources and Referrals by Domain 20](#_Toc193884192)

[8. Patient-Level Data Page 20](#_Toc193884193)

[9. The ACORN Map 21](#_Toc193884194)

[Frequency Asked Questions (FAQ) 22](#_Toc193884195)

[ACORN Dashboard Data Definitions Table 25](#_Toc193884196)

**For questions, concerns, or troubleshooting support about the ACORN Dashboard, contact the ACORN Dashboard Team at** [**VHAACORNDASHBOARD@va.gov**](mailto:VHAACORNDASHBOARD@va.gov)**.**

# **Revision History**

| Date | Version Name | Description | Authors |
| --- | --- | --- | --- |
| Feb. 2024 | 1.1 | Published user guide and FAQ | Sarah M. Leder  Lauren E. Russell  Kathleen M. Mitchell  Meaghan A. Kennedy |
| Nov. 2024 | 2.0 | Addition of Patient-Level Data and Map pages to ACORN Dashboard, general updates | Sarah M. Leder  Kathleen M. Mitchell  Lauren E. Russell |
| March 2025 | 2.1 | General updates | Kathleen M. Mitchell |

# **ACORN Screening Tool**

## Introduction

Assessing Circumstances and Offering Resources for Needs (ACORN) is a standardized approach to identifying and addressing health-related social needs among Veterans receiving care in the Veterans Health Administration (VHA). To enhance existing VHA screening processes and interventions, the ACORN model consists of a standardized screening tool and the provision of relevant resources and referrals to help address Veterans’ identified needs.

ACORN aims to: 1) systematically screen Veterans for health-related social needs at the point of care; 2) provide clinical care teams real-time information about Veterans’ unmet needs; and 3) address identified needs by offering referrals to Social Work, Mental Health, or other relevant VA services; support navigating VA and community resources; and/or geographically tailored resource guides.


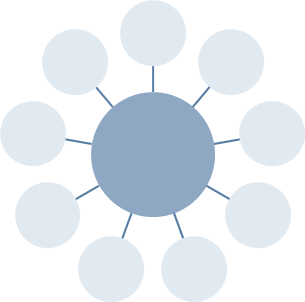

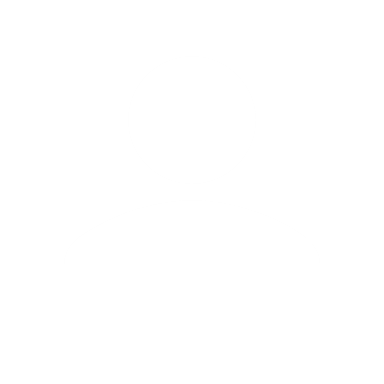

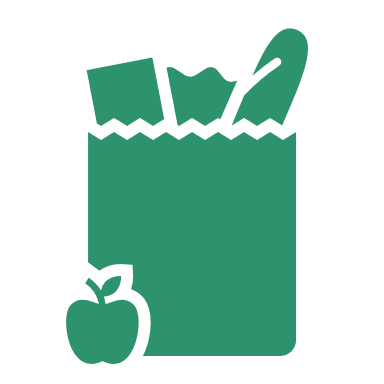

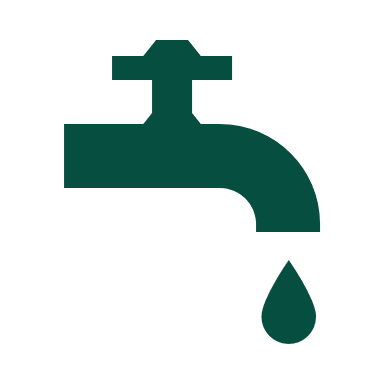

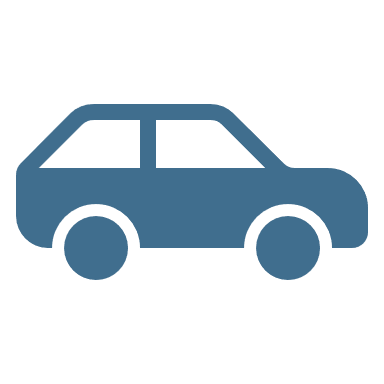

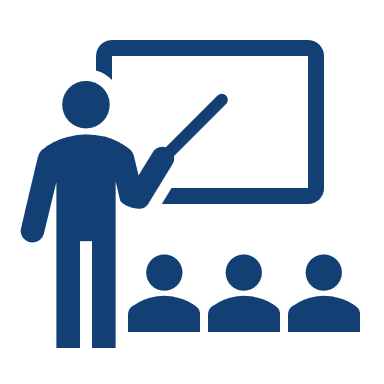

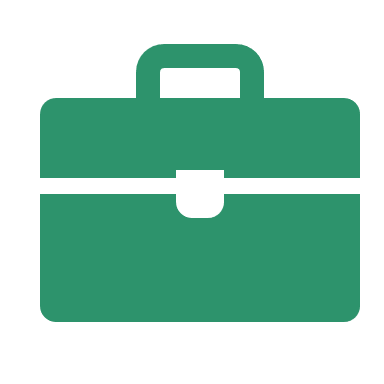

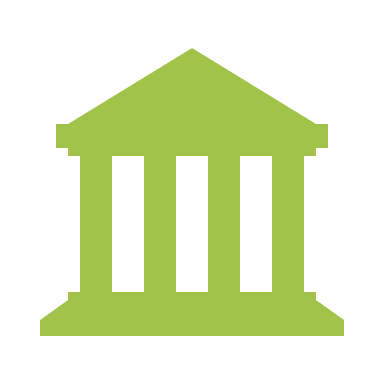

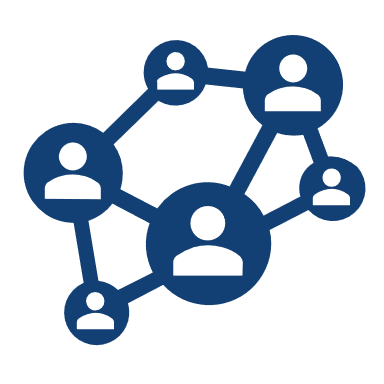

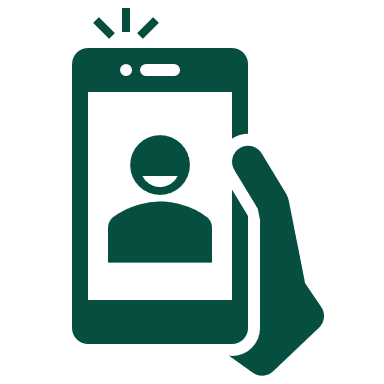

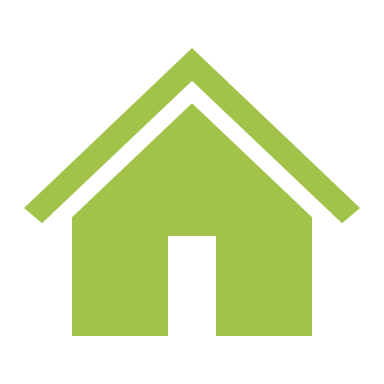


ACORN screens across nine domains, including: food, housing, utilities, transportation, education, employment, legal, social isolation/loneliness, and digital needs (device/internet access and digital health literacy).

The existing national VHA clinical reminders for housing instability and food insecurity are integrated into the ACORN screening tool, meaning answering these questions as part of ACORN satisfies the clinical reminder requirements.

In 2018, an interprofessional team of clinical leaders, staff physicians, social workers, mental health providers, informaticists, researchers, and other subject matter experts in the VA New England Healthcare System (VISN 1), developed the original ACORN screening tool and resource guides.^1^ Partnerships with the Office of Health Equity began in 2019 and with the National Social Work Program in 2021. ACORN has since been implemented in a variety of clinical settings, including Primary Care (Patient-Aligned Care Teams [PACT]), Mental Health, Geriatrics, Women’s Health, and a range of specialty clinics, as well as in emergency departments and inpatient settings.^2^

### Additional ACORN Information

Visit the ACORN SharePoint *[internal to VHA staff only]* for additional program information and resources, such as the ACORN Screening Tool Overview *[internal to VHA staff only]* and CPRS Template PowerPoint *[internal to VHA staff only]*. For a comprehensive overview of the ACORN pre-implementation process and site expectations, review the ACORN Implementation Toolkit *[internal to VHA staff only]* and Getting Started Checklist *[internal to VHA staff only]*.

The ACORN Leadership Team is available to support VHA facilities and clinical care teams interested in implementation at [VHAACORN@va.gov](mailto:VHAACORNDASHBOARD@va.gov).

1. *Russell, L.E.*, Cohen, A.J.* (*co-first authors), Chrzas, S. et al. Implementing a Social Needs Screening and Referral Program Among Veterans: Assessing Circumstances & Offering Resources for Needs (ACORN). J GEN INTERN MED 38, 2906–2913 (2023). https://doi.org/10.1007/s11606-023-08181-9*
2. *Cohen AJ, Russell LE, Elwy AR, Mitchell KM, Cornell PY, Silva JW, et al. Adaptation of a social risk screening and referral initiative across clinical populations, settings, and contexts in the Department of Veterans Affairs Health System. Frontiers in Health Services. 2023 2023-January-30;2.*

**Positive Screening Results**

The following questions are included on the ACORN screening tool. Responses highlighted in green indicate a “positive” need that warrants follow-up care.

| 1. **In the past two months, have you been living in stable housing that you own, rent, or stay in as part of a household?^1^** | | | | |
| --- | --- | --- | --- | --- |
| 1. Yes – Living in stable housing | | | | |
| ** (1.1) Are you worried or concerned that in the next two months you may NOT have stable housing  that you own, rent, or stay in as part of a household?^1^**   1. *Yes – worried about housing near future* | | | | |
| ** (1.2) Where have you lived for MOST of the past two months?^1^** | | | |  |
| 1. Apartment/House/Room (no government subsidy) 2. Apartment/House/Room (with government subsidy) 3. With Friend/Family 4. Motel/Hotel | | 1. Short-term Institution like Hospital,  Rehab Center, Drug Treatment Center 2. Homeless Shelter 3. Anywhere outside (e.g., Street, Vehicle,  Abandoned Building) 4. Other | |  |
| 1. *No – Not worried about housing near future* | | | |  |
| 1. No – Not living in stable housing | | | | |
| ** *Collect answer for the question “Where have you lived for MOST of the past two months?” ^1^*** | | | | |
| - *If respondent endorses either “not living in stable housing” OR “worried about housing near future” for (1):*   **(1.3) Are you currently without a place to stay?** | | | | |
| 1. Yes | | 1. No | | |
| 1. I’m going to read you two statements that people have made about their food situation. For each statement, please tell me  whether the statement was often true, sometimes true, or never true for your household in the last 12 months.   **(2.1) Within the past 12 months, you worried whether your food would run out before you got money to buy more.^2^** | | | | |
| 1. Often true | | 1. Sometimes true | | 1. Never true |
| **(2.2) Within the past 12 months, the food you bought just didn’t last and you didn’t have money to get more.^2^** | | | | |
| - 1. Often true | | - 1. Sometimes true | | - 1. Never true |
| - *If respondent endorses “often true” or “sometimes true” for either “food would run out” (2.1) OR “food didn’t last”  (2.2):*   (2.3) Do you need help getting food for this week? | | | | |
| 1. Yes | | 1. No | |  |
| 1. **How often do you have trouble paying for your utilities (e.g., electric, gas, oil, water, or phone)?^3^** | | | | |

| - 1. Often | - 1. Sometimes | - 1. Never | - 1. Not applicable/I don’t pay for utilities |
| --- | --- | --- | --- |

| - *If respondent endorses “often” or “sometimes” for (3):*   (3.1) Has the electric, gas, oil, water or phone company threatened to shut off services in your home?^4^ |
| --- |

| 1. Yes | 1. No | 1. Already shut off | 1. Not applicable/I don’t pay for utilities |
| --- | --- | --- | --- |

| 1. **How often has lack of transportation kept you from medical appointments, meetings, work, or from getting things needed for daily living?^5^** |
| --- |

| - 1. Often | - 1. Sometimes | - 1. Never |
| --- | --- | --- |

| - *If respondent endorses “often” or “sometimes” for (4):*   (4.1) Do you need assistance with transportation for an upcoming appointment? | |
| --- | --- |
| 1. Yes | 1. No |
| 1. **Do you currently have any legal matters you need help with (e.g., child support or custody, divorce, debt or credit problems, or need for a discharge upgrade)?** | |
| 1. Yes | 1. No |
| 1. **How often do you feel lonely or isolated from those around you?^6^** | |

| - 1. Often | - 1. Sometimes | - 1. Never |
| --- | --- | --- |

| 1. **Do you want help finding or keeping work or a job?^7^** | | |
| --- | --- | --- |
| \| - 1. Yes, help finding work \| - 1. Yes, help keeping work \| - 1. No, I don’t want help finding or keeping work \| \| --- \| --- \| --- \| | | |
| 1. **Do you want more information about educational benefits and resources for Veterans?** | | |
| 1. Yes | 1. No | |
| 1. **Do you have access to any of the following devices? (Please select all that apply.)** | | |
| - Landline - Simple cell phone (flip phone) - Smartphone (a cell phone with a touch screen and internet) | | - Computer (laptop, desktop, or tablet such as an iPad) - None |
| 1. **Do you have access to affordable and reliable internet where you live?** | | |

| - 1. Yes | - 1. No | - 1. Not applicable/I don’t want internet access |
| --- | --- | --- |

| 1. **Would you like help learning to use a smartphone, tablet, or computer to access VA healthcare online (e.g., video visits, medical record, secure messaging)?** |
| --- |

| 1. Yes | 1. No | 1. Not applicable/I don’t have any of these devices |
| --- | --- | --- |

*When derived from the* [Centers for Medicare and Medicaid Services (CMS) Accountable Health Communities (AHC) Screening Tool](https://innovation.cms.gov/files/worksheets/ahcm-screeningtool.pdf)*, the original source is cited per* [AHC guidance](https://innovation.cms.gov/media/document/ahcm-screening-tool-citation)*. Questions without citations were developed by the VHA ACORN team and collaborators across multiple VHA offices and sites.*

1. VA National Center on Homelessness Among Veterans. “Homeless Screener.” U.S. Department of Veterans Affairs, September 2020. https://www.va.gov/HOMELESS/nchav/resources/prevention/homeless-screener.asp
2. Hager, E. R., Quigg, A. M., Black, M. M., Coleman, S. M., Heeren, T., Rose-Jacobs, R., Cook, J. T., Ettinger de Cuba, S. E., Casey, P. H., Chilton, M., Cutts, D. B., Meyers A. F., Frank, D. A. (2010). Development and Validity of a 2-Item Screen to Identify Families at Risk for Food Insecurity. Pediatrics, 126(1), 26-32. doi:10.1542/ peds.2009-3146.
3. Adapted with permission from Page-Reeves J, Kaufman W, Bleecker M, Norris J, McCalmont K, Ianakieva V, Ianakieva D, Kaufman A. Addressing Social Determinants of Health in a Clinic Setting: The WellRx Pilot in Albuquerque, New Mexico. J Am Board Fam Med. 2016 May-Jun;29(3):414-8. doi: 10.3122/jabfm.2016.03.150272. PMID: 27170801.
4. Adapted with permission from Cook, J. T., Frank, D. A., Casey, P. H., Rose-Jacobs, R., Black, M. M., Chilton, M., . . . Cutts, D. B. (2008). A Brief Indicator of Household Energy Security: Associations with Food Security, Child Health, and Child Development in US Infants and Toddlers. Pediatrics, 122(4), 867-875. doi:10.1542/peds.2008-0286.
5. Adapted with permission from the national PRAPARE® social determinants of health protocol developed by the National Association of Community Health Centers, the Association of Asian Pacific Community Health Organizations, and the Oregon Primary Care Organization and their development partners. www.nachc.org/prapare. ©National Association of Community Health Centers. All Rights Reserved.
6. Adapted with permission from Anderson, G. Oscar and Colette E. Thayer. Loneliness and Social Connections: A National Survey of Adults 45 and Older. Washington, DC: AARP Research, September 2018. https://doi.org/10.26419/res.00246.001
7. Identifying and Recommending Screening Questions for the Accountable Health Communities Model (2016, July) Technical Expert Panel discussion conducted at the U.S. Department of Health and Human Services, Centers for Medicare & Medicaid Services, Baltimore, MD.

## Disposition Field / Action Steps

A list of standard disposition fields or “action steps” is included at the end of the CPRS template. Staff will use this field to note which resources and referrals, if any, were provided to the Veteran the day of screening to address needs identified. Action Steps include providing resource information or guides, warm hand-offs or consults to Social Work, and referrals to other VHA and community services.

Similar to the response options for the social need domains, the options provided in the disposition section are linked to health factors. The resources and referrals data included in the ACORN Dashboard uses the health factors embedded in the disposition field.

For additional guidance on using the disposition section, review the ACORN CPRS Disposition Section Recommendations handout *[internal to VHA staff only]*.

### Disposition Field / Action Steps in the ACORN CPRS Template


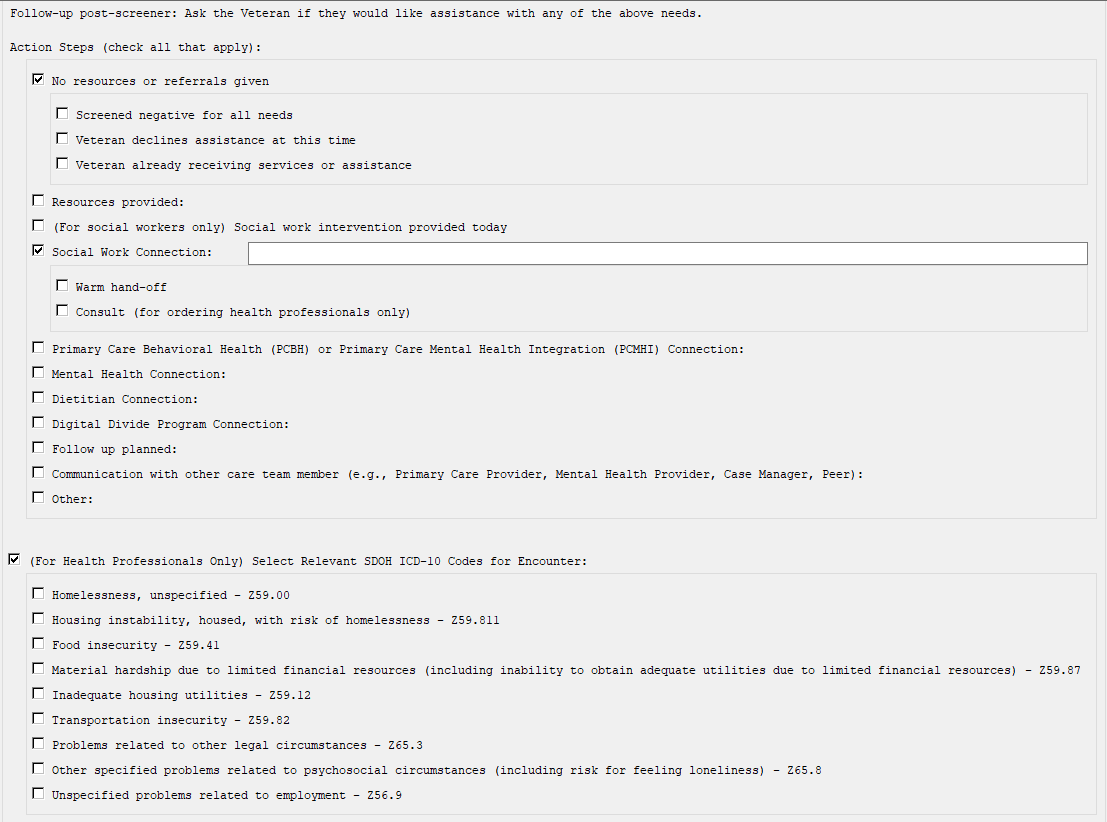


# **ACORN Dashboard**

## Purpose and Rationale

The ACORN Dashboard was created in 2023 to increase access to data collected through the ACORN screening tool. This Dashboard will help VHA clinical care teams and programs: 1) understand the health-related social needs impacting Veterans; 2) track the types of resources and referrals provided to Veterans who screen positive on ACORN; and 3) observe how screening rates and resources and referrals vary by demographic factors and over time.

The *Patient-Level Data page* was added to the ACORN Dashboard in 2024 to support care teams
in better understanding individual Veterans’ endorsed needs in their clinical setting, tracking the resources and referrals provided to each Veteran, and facilitating follow-up opportunities.

| **All VHA employees can access the Dashboard on the ACORN SharePoint *[internal to VHA staff only]* or directly through this link *[internal to VHA staff only]*.** |
| --- |

## Data Description

Dashboard data are updated twice daily at 9:30am and 1:00pm ET.

The ACORN CPRS Template includes embedded health factors. Responses captured in the template are pulled from the VHA Corporate Data Warehouse (CDW). The use of health factors facilitates evaluation of screening results and the interventions provided to address identified needs at the national, VISN, facility, and clinic level. A combination of administrative data from the CDW and health factors are used to populate the Patient-Level Data page.

On pages with calculated percentages, the percentage of positive screens is calculated using the total number of ACORN screens as a denominator. The percentages of “Any Resource or Referral,” “Already Receiving Assistance,” and “Declined Assistance” are calculated using the number of positive ACORN screens as a denominator.

## Target Audience

The ACORN Dashboard aims to meet the needs of care team members, including physicians, nurses, dietitians, social workers, peer specialists, Whole Health partners and coaches, and VHA leaders.

## A Note on Protected Health Information

***Protected Health Information (PHI) is only accessible on the Patient-Level Data page of
the ACORN Dashboard.***

The primary function of the Dashboard is to display aggregate data at the national level. This means that the following pages will not contain PHI, and it is not possible to view or retrieve patient-specific data:

- Overview Data
- Demographics
- Domain Trends
- Resources and Referrals
- Co-Occurring Needs by Domain
- Resources and Referrals by Domain
- Patient-Level Data

For these pages, ACORN Screens is an umbrella category and denotes that the screening was completed, so will display numbers fewer than 13 (screenshot below).

However, to protect Veteran privacy, Positive ACORN Screens, Any Resource or Referral, Already Receiving Assistance, and Declined Assistance counts are hidden in the ACORN Dashboard where totals are fewer than 13 (“<13” will appear in place of a value). An example of this feature is provided below, where the number of Veterans across the age groups who declined assistance were fewer than 13.


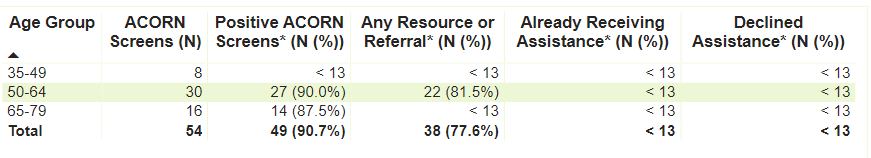


If no screens were completed for a particular demographic group in the facility or setting selected, the group will not appear on the Dashboard tables. The example above shows a facility or setting in which no Veterans between ages 18-34 or 80+ have been screened, so the respective rows for those categories are currently hidden in the table.

In the 2024 update, a Patient-Level Data page was added to allow end users to view patient-level information. For additional information on the data available for this page, visit the Patient-Level Data Page section.

## Data Definitions

### Summary Tables on National-Level Pages


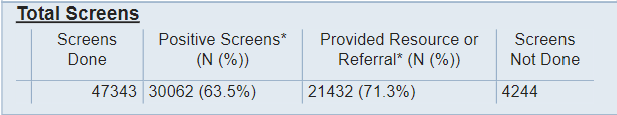


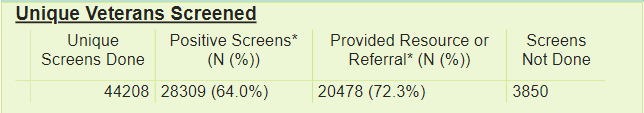


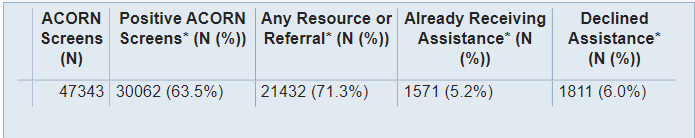


*ACORN Screens*

The number of ACORN Screens completed based on any ACORN health factor captured in CDW. This number will typically be higher than the number of unique Veterans screened because individual Veterans may be screened more than once during the period shown.

*Positive ACORN Screens*

The number of ACORN Screens that include at least one positive response across the nine domains. The percentage of positive ACORN screens is based on the number of Positive ACORN Screens divided by the number of ACORN Screens completed.

*Any Resource or Referral*

The number of Positive ACORN Screens for which a resource or referral options is selected in the Disposition Section/Action Steps section of the template. The percentage is based on the number of Positive ACORN Screens associated with any resource or referral divided by the number of Positive ACORN Screens.

The list of resource or referral options included in the disposition section include: Resources provided; (For social workers only) Social work intervention provided today; Social Work Connection; Primary Care Behavioral Health (PCBH) or Primary Care Mental Health Integration (PCMHI) Connection; Mental Health Connection; Dietitian Connection; Digital Divide Program Connection; Follow up planned; Communication with other care team member (e.g., Primary Care Provider, Mental Health Provider, Case Manager, Peer); and Other).

*ACORN Screens Not Done*

The total number of instances in which staff selected “Screening not performed” on the CPRS template. This number does not account for all eligible Veterans; thus, it likely undercounts the number of total Veterans eligible for ACORN who were not screened.


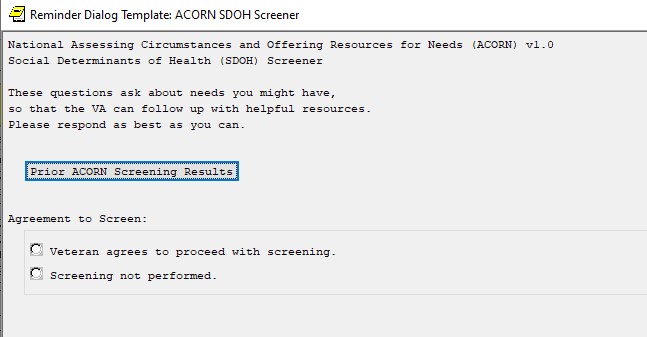

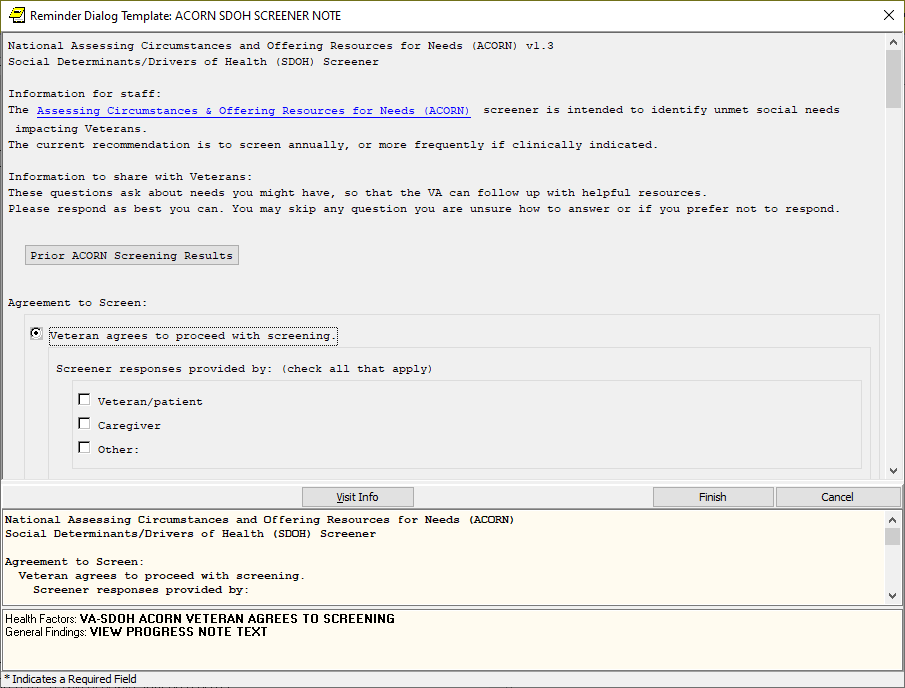


*Already Receiving Assistance*

The number of Positive ACORN Screens for which “Veteran already receiving services or assistance” is selected in the disposition section of the template. This response option indicates the Veteran had a positive ACORN screen but reported they were already receiving assistance at the time of screening. The percentage is based on the number of Positive ACORN Screens associated with “Already receiving assistance” divided by the total number of positive ACORN Screens.

*Declined Assistance*

The number of Positive ACORN Screens for which “Veteran declines assistance at this time” is selected in the Action Steps section of the template. This response option indicates that the Veteran had a positive ACORN screen but declined assistance (resources and/or referrals) at the time of the screen. The percentage is based on the number of Positive ACORN Screens associated with “Declined assistance” divided by the total number of Positive ACORN Screens.


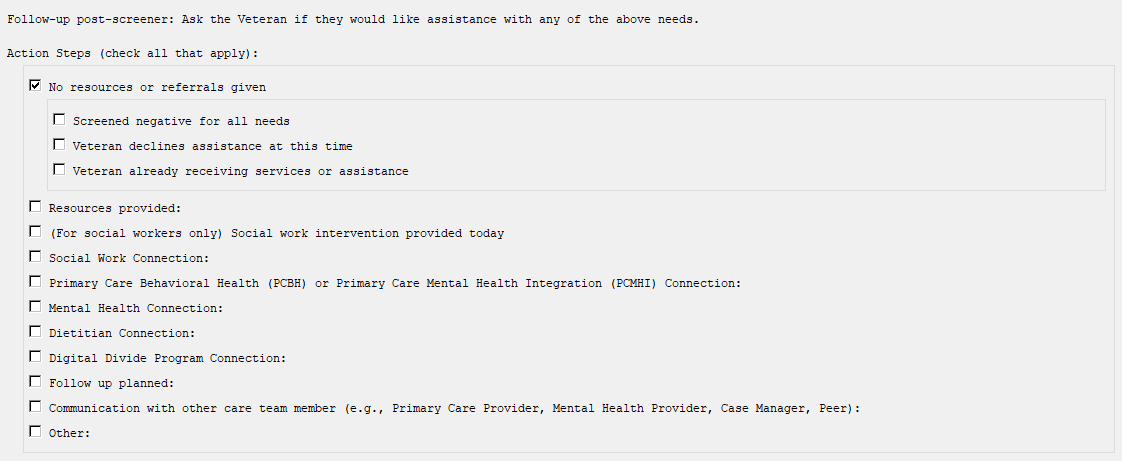


# **How to Use the ACORN Dashboard**

## On-Screen Filters and Tooltip Feature


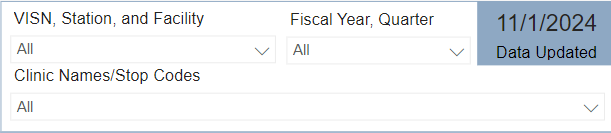


There are currently five filters and one search bar used throughout the Dashboard:

- VISN (Veterans Integrated Service Network)
- Facility Name
- Station Name
- Fiscal Year (October 1st-September 30th)
- Clinic Names/Stop Codes
- Patient-Level Data Table Search *[available only on the Patient-Level Data page]*

To select your VISN, Facility, and Station, use the down arrow to view the dropdown menu and
click the relevant checkboxes for VISN, facility, and/or station.

To select the desired Fiscal Year and Quarter, use the down arrow to view the dropdown menu
then click the relevant checkboxes.

*Clinic Names/Stop Codes Filter*

The ‘Clinic Names/Stop Codes’ filters for specific stop codes or categories of care. The included clinic names and stop codes are based on those attached to the ACORN-associated encounter. Only the clinic name and stop code combinations used when the ACORN screening was completed will populate in the Dashboard, meaning some clinic names, stop codes, and stop code combinations may not appear in the list.

To select a clinic name and/or stop code, use the down arrow to view the dropdown menu then click the relevant checkboxes. Clicking on the "Clinic Names/Stop Codes" filter will also populate a search function at the top of the dropdown list to search types of care or specific stop codes. Given the volume or clinic names and stop codes in VA, the filter is based on stop code classes.

*Note:* the relevant class information is missing for some clinics and stop codes. In those instances, the
stop code name (instead of the class name) is used.

*Reset Feature*


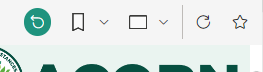
Applied filters are carried over each page. These filters will also remain applied even when leaving and reopening the Dashboard. If the counterclockwise arrow in the upper right corner of the Dashboard toolbar is green, then the filters, slicers, or other data view changes are still applied.

To remove all filters and other changes, click the counterclockwise arrow (outlined in orange) to reset the Dashboard to default settings.

*Tooltip Feature*

When viewing the graphs and tables on the Dashboard, allowing the mouse to hover over any point or bar on a graph will make a dark grey ‘tooltip’ box appear with additional information. Below are two examples of the tooltip feature.


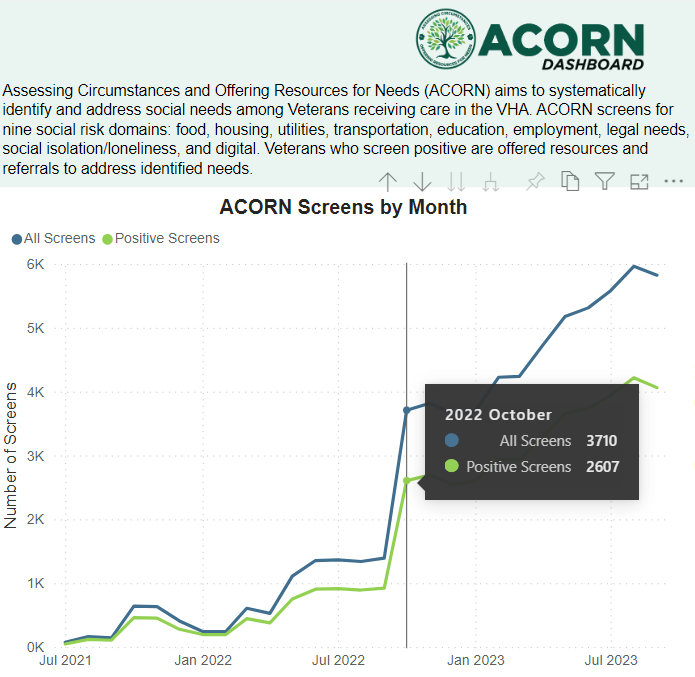

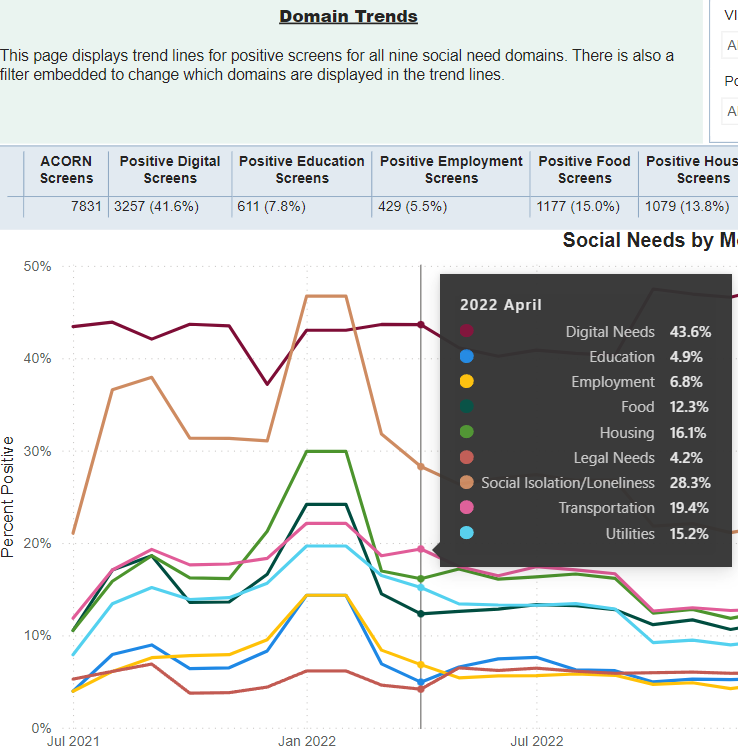


## Patient-Level Data Page Table Search

Available only on the Patient-Level Data page, staff can select one of the four categories in the Patient-Level Data Table Search to filter patient information.


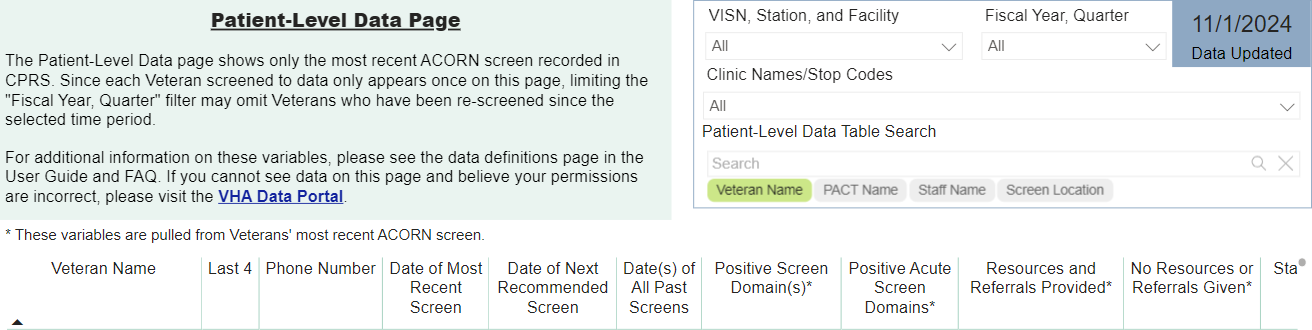


Users can use the four categories to search for specific information on the Patient-Level Data page:

- *Veteran Name:* The default search option. To search for a specific Veteran, enter their name into the search box beginning with last name. Veterans’ names are formatted on the Dashboard as Last Name, First Name, Middle Initial (if applicable).
- *PACT Name:* Select this option to search for screens completed in a specific PACT setting.
- *Staff Name:* Select this option to search by staff names of those who completed an ACORN screen with a Veteran. Associated staff names are populated by who created the ACORN screen in CPRS.
- *Screen Location (primary stop code class):* Select this option to search by primary class in the stop code hierarchy. This option mirrors the VSSC Appointments report *[internal to VHA staff only]*. Additional information can be found in the VSSC Access Team’s Data Definitions document *[internal to VHA staff only]*.


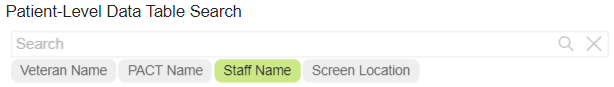
When a category is selected, its box will turn green. An example of a category being selected is shown on the right.

## ACORN Implementation Map

Each circle on the map represents a unique clinical setting, defined by stop codes. If the screening tool is being used in more than one setting per location, multiple colors will be shown in the circle. The portion of each circle does not correlate to the number of screens administered in that specific clinical setting.


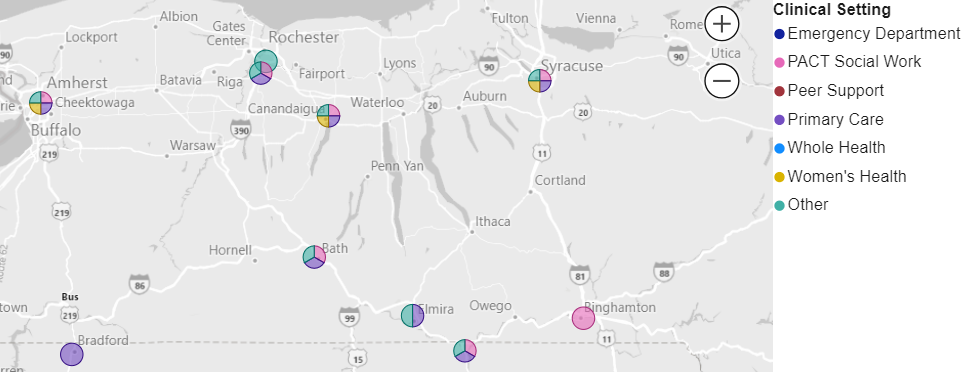


## Exporting Information from the Dashboard

The ribbon at the top of the Dashboard has icons on the left and on the right.


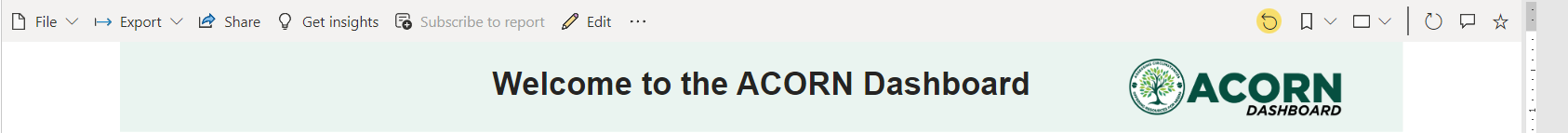

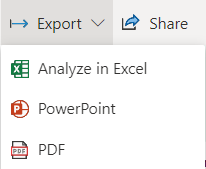


Clicking on the second option from the left, **‘Export’**, will open a dropdown. Staff are able to select one of the following two for exporting data from the Dashboard: PowerPoint and PDF. See steps below.

*Note:* The Analyze in Excel feature is not enabled for the ACORN Dashboard. The Share button is only available if you have access to PowerBI Pro.

*The Export Pop-Up Window*

On this pop-up, users can specify which pages and values they wish to export.


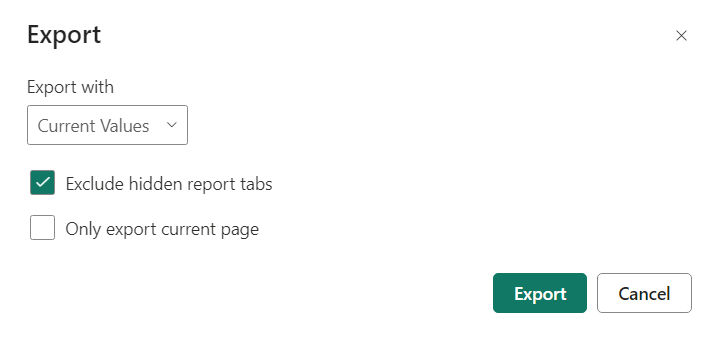
Export with…

- *Current Values:* download pages with all filters that are currently applied to the Dashboard.
- *Default Values:* download pages with the default view. The default view includes all VISNs, Facilities, Stations, Fiscal Years, and Quarters.

‘Exclude hidden reports tabs’ checkbox is automatically selected. Unchecking this option will export all pages of the Dashboard at once, including multiple demographics and domains pages.

‘Only export current page’ checkbox is automatically de-selected (by PowerBI). Checking this option will only export the page that is currently open.Pages on the ACORN Dashboard

This chapter will detail the pages available on the ACORN Dashboard.

## 1. Before You Begin

This page provides a tutorial to orient users to the ACORN Dashboard, navigation, and page contents.


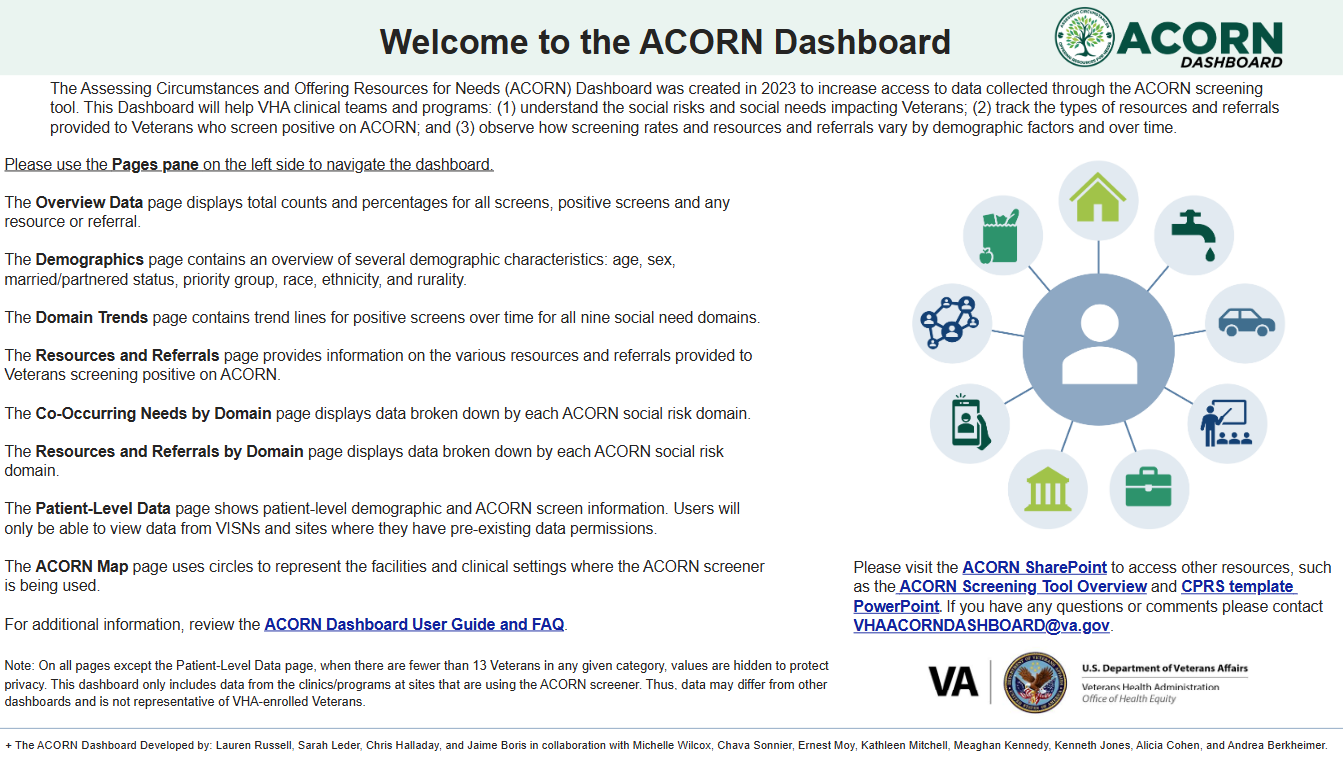


## 2. Overview Data

The Overview Data page displays total counts and percentages for all ACORN Screens completed as well as unique Veterans screened, including corresponding Positive ACORN Screens, Any Resource or Referral, and ACORN Screens Not Done. Additionally, trend lines depict the number of all screens and positive screens over time. The bar graph displays the percentages of Veterans screened positive for each health-related social need domain on the ACORN screening tool.


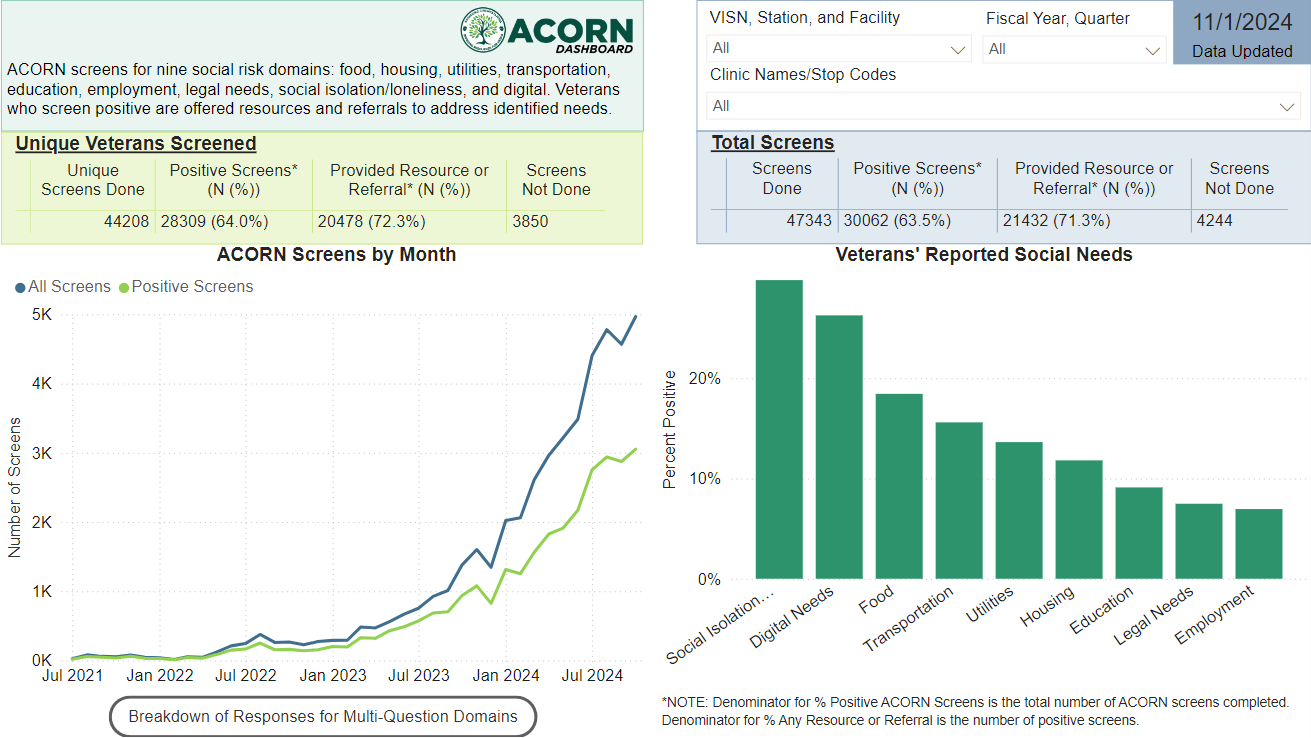


Selecting the “Breakdown of Responses for Multi-Question Domains” button below the trend lines graph (outlined in orange) will replace the trend lines with a table displaying the number of responses for each domain with more than one question on the ACORN screening tool.


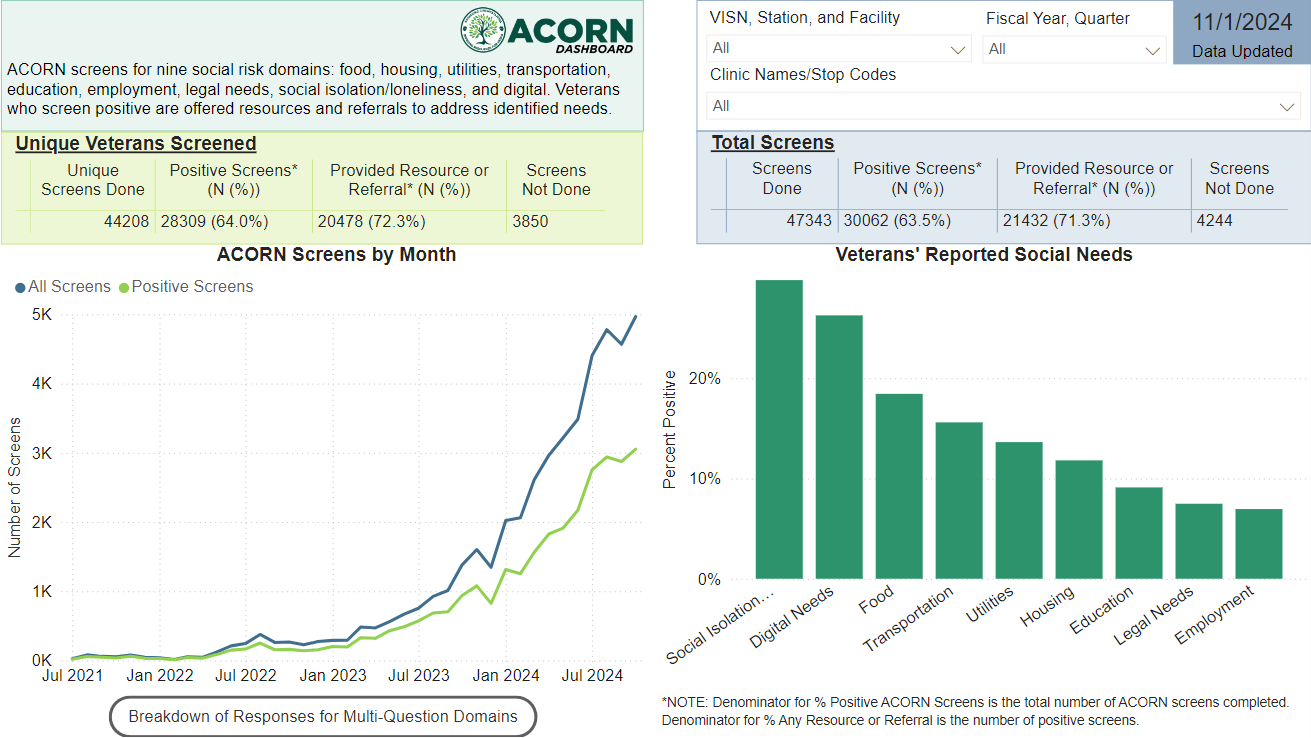


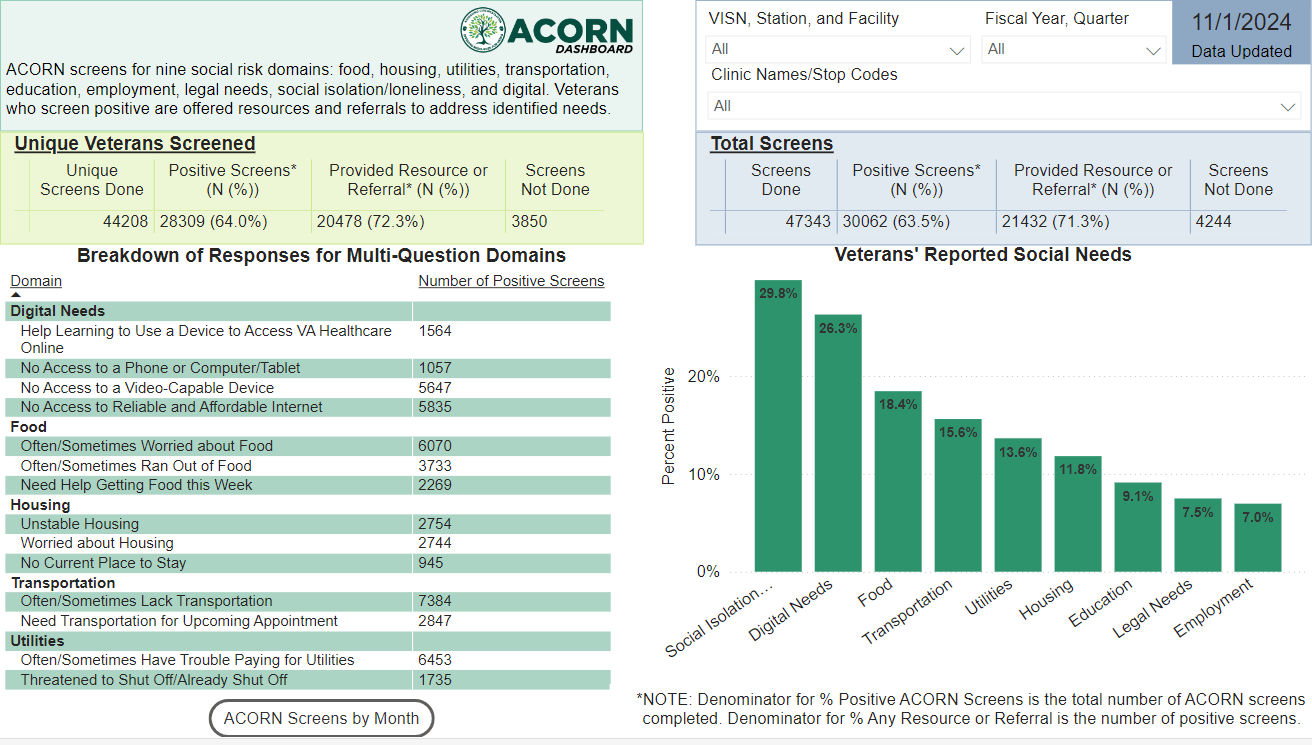


To return to the trend lines, select the “ACORN Screens by Month” button below the table (outlined in blue).

For instructions on how to filter national-level ACORN data to specific VISN, facility, and/or station-level data, refer to the How to Use the ACORN Dashboard chapter.

## 3. Demographics

The Demographics Page shows ACORN screening data broken down by specific demographic characteristics. Below are the demographic characteristics descriptions.


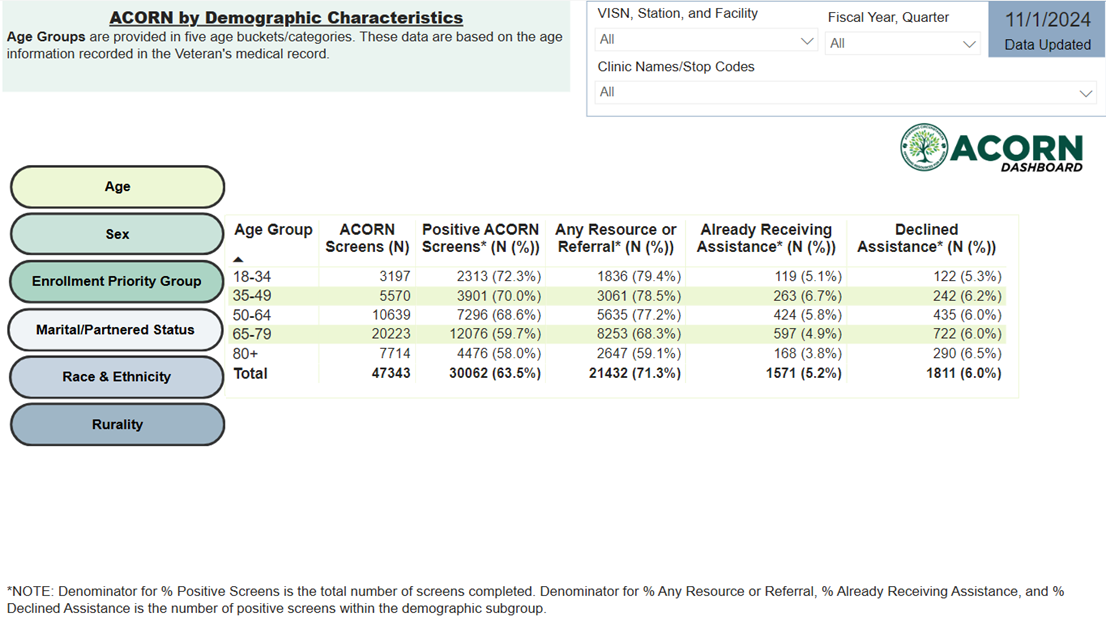

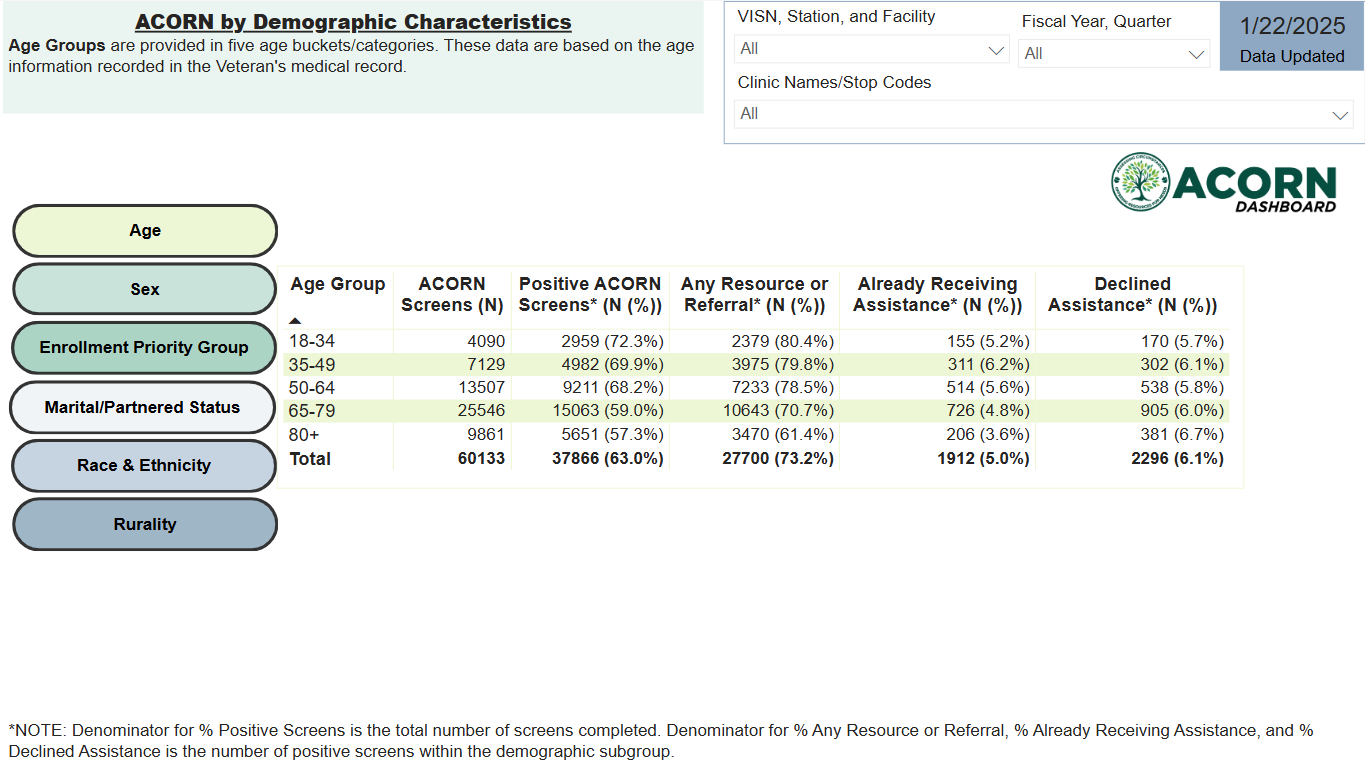

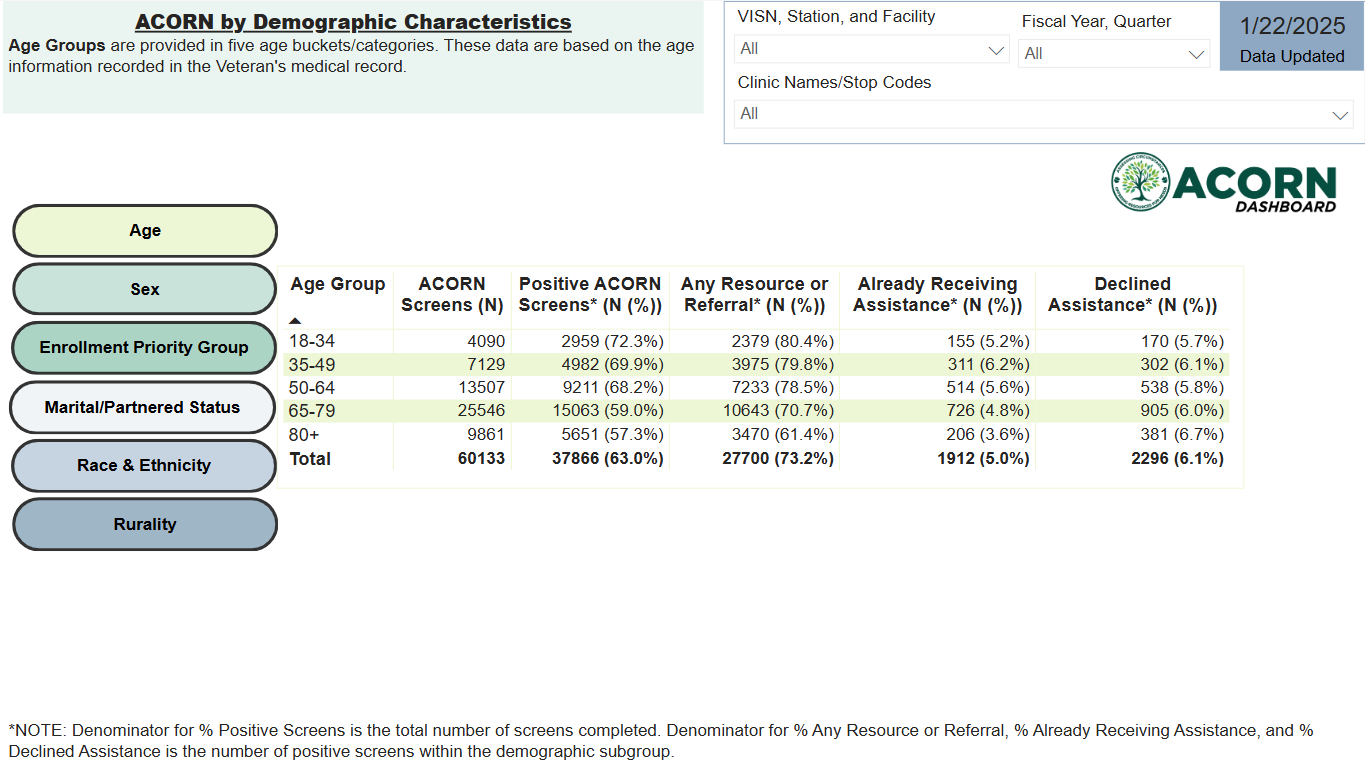

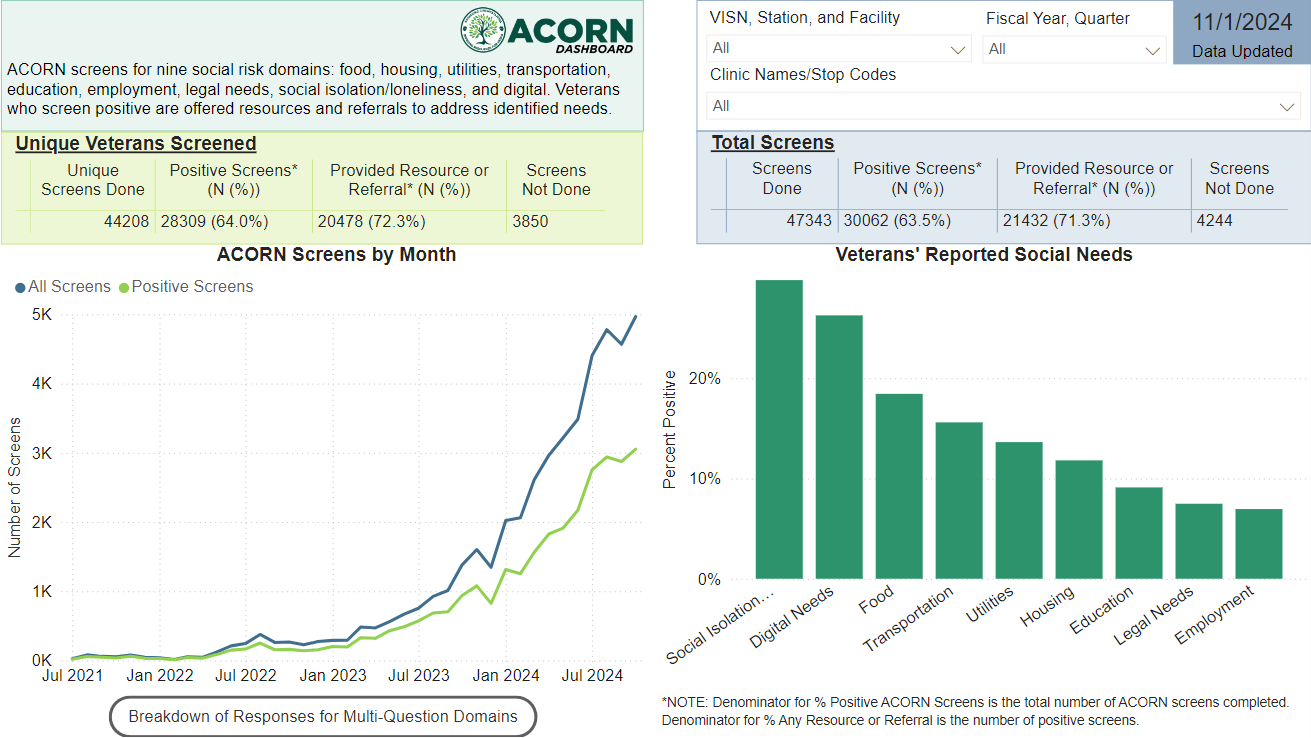


*Age*

Age groups are displayed in 15-year ranges and based on the Veteran’s age at time of ACORN screening.

*Sex*

Sex refers to the classification of individuals as female or male. In VA medical records, this information is typically the sex recorded on the Veteran’s original birth certificate.

*Enrollment Priority Group*

Enrollment Priority Group refers to the determination of a Veteran's eligibility for and cost-share associated with VA health benefits and service-connected disability compensation. Enrollment Priority Groups (numbered 1-8) are assigned by VA during Veteran enrollment.

To learn more about Enrollment Priority Groups, visit the [VA Priority Groups webpage](https://www.va.gov/health-care/eligibility/priority-groups/).

*Married and Partnered Status*

Married/Partnered status refers to the Veteran's relationship status, based on the responses recorded in the Veteran’s medical record.

*Race and Ethnicity*

Race and ethnicity are based on the racial and ethnic groups approved by the Office of Management and Budget (OMB) and currently used in the VA. Race and ethnicity are combined in the ACORN Dashboard per the Office of Health Equity’s approach to race and ethnicity categorization. For example, any Veteran who identifies as Hispanic or Latino appears only in that category and not in a race category, even if a race was selected in addition to Hispanic and Latino ethnicity in the Veteran’s medical record.

For more information about OMB’s standards, review the [OMB’s Statistical Policy Directive (SPD) No. 15 Memorandum](https://www.federalregister.gov/documents/2024/03/29/2024-06469/revisions-to-ombs-statistical-policy-directive-no-15-standards-for-maintaining-collecting-and) or the [OMB’s Notice of Decision: Revisions to the Standards for the Classification of Federal Data on Race and Ethnicity](https://www.govinfo.gov/content/pkg/FR-1997-10-30/pdf/97-28653.pdf).

*Note*: VHA is developing a plan to incorporate OMB’s 2024 updates, and the Dashboard will incorporate these changes once implemented. While SPD 15 is effective immediately, federal agencies have until March 28, 2029 to bring existing data collection and reporting activities into compliance, and must submit action plans to OMB by September 28, 2025.

*Rurality*

Rurality refers to the Veteran’s rurality based on the most recent address listed in their medical record. These data are based on the Rural-Urban Commuting Areas (RUCA) System developed by the U.S. Department of Agriculture and the Department of Health and Human Services. There are three categories: urban, rural, and highly rural. To learn more about how VA categorizes rurality, visit the [Office of Rural Health webpage](https://www.ruralhealth.va.gov/aboutus/ruralvets.asp).

## 4. Domain Trends

The Domain Trends page contains trend lines for positive screens over time for all nine domains.


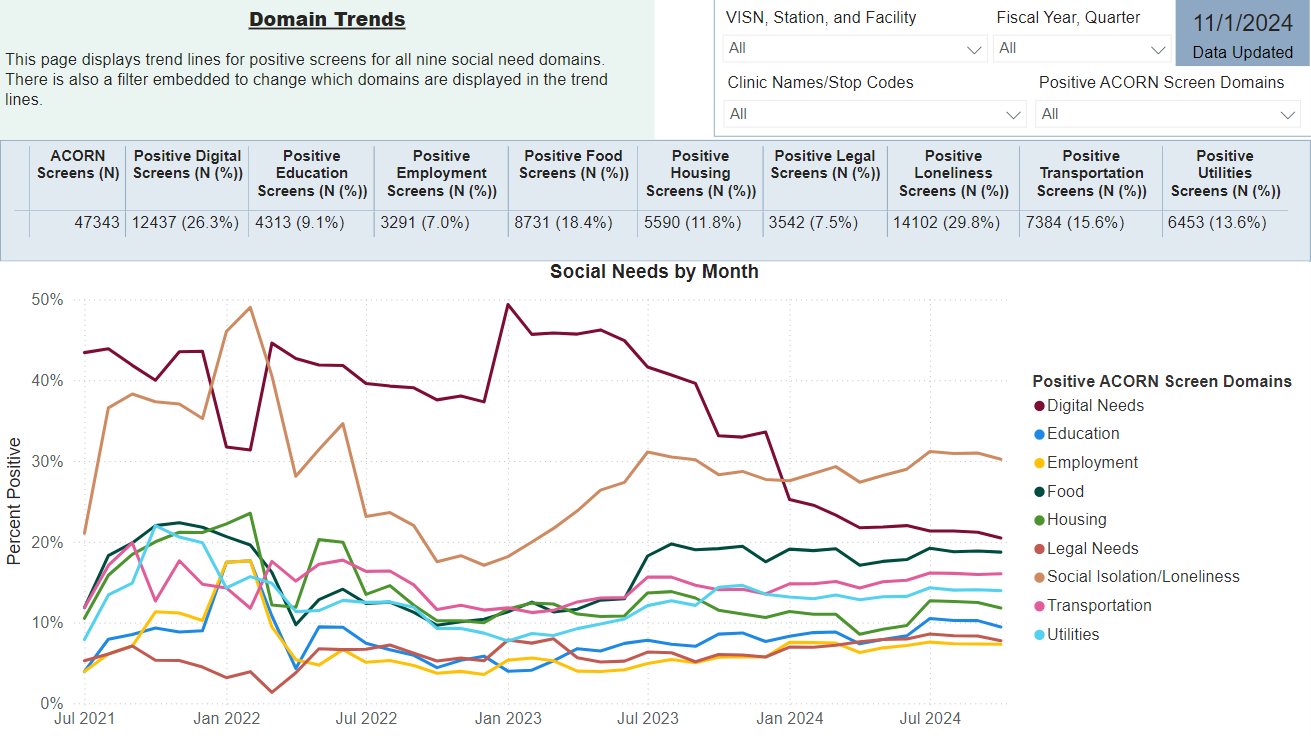


## 5. Resources and Referrals

The Resources and Referrals page displays the breakdown of resources and referrals provided to Veterans based on the options selected in the Disposition Field/Action Steps section. The table shows the number and percentage of positive screens for which Any Resource or Referral was provided and for which the Veteran reported they were Already Receiving Assistance or Declined Assistance.


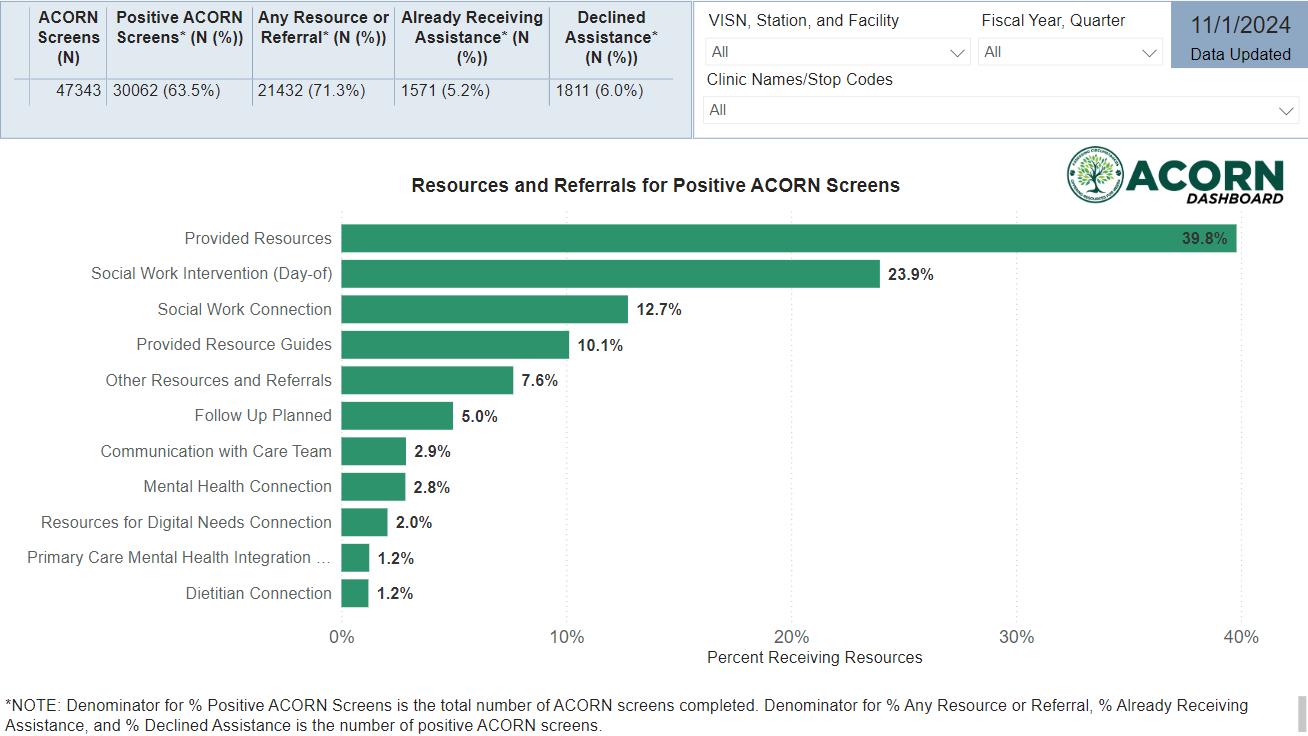


## 6. Co-Occurring Needs by Domain

The Co-Occurring Needs by Domain page provides information on co-occurring positive domains. In the table, the domain-specific positive screens count is based on the number of screens where the Veteran endorsed a need in the selected domain either alone or in combination with other unmet needs. The graph shows the overlap between the selected domain (in this case, Digital Needs) and the other eight domains.


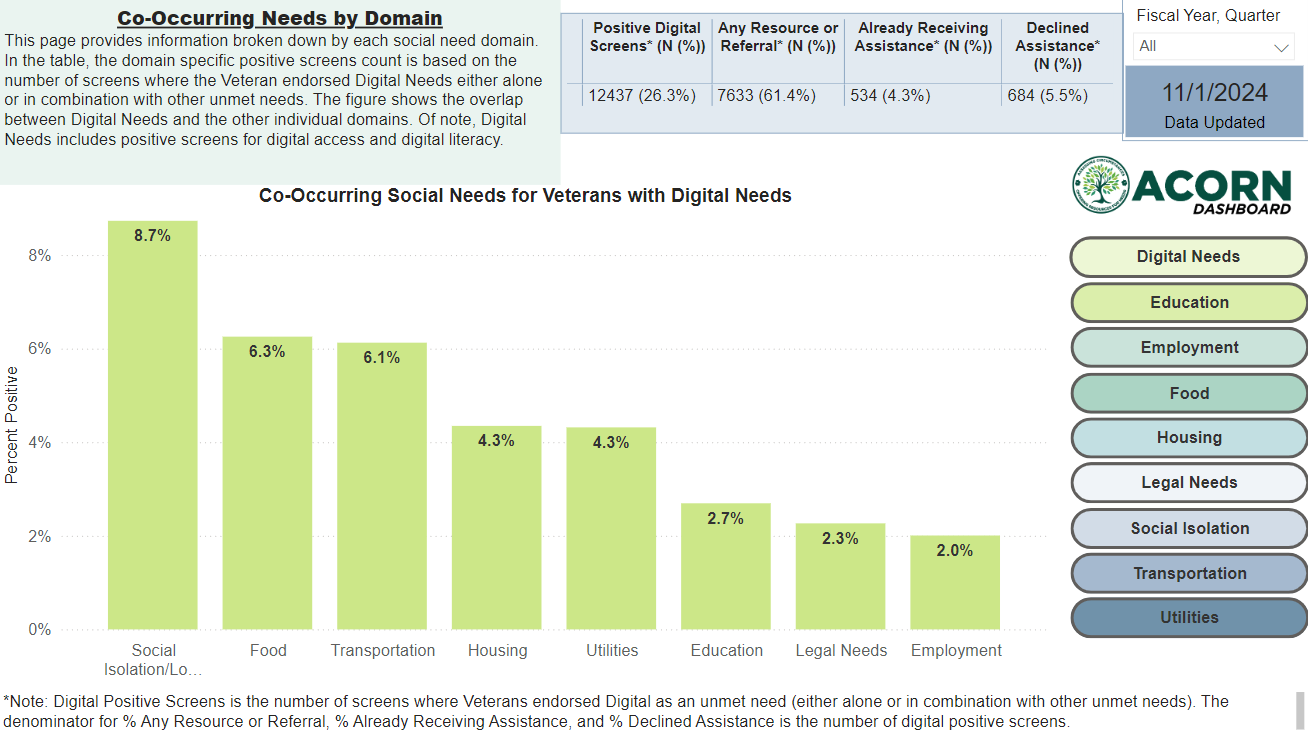


## 7. Resources and Referrals by Domain

The Resources and Referrals by Domain page provides information broken down by each domain. The graph shows the resources and referrals provided to Veterans who screened positive for the selected domain and/or another reported unmet need at the time of screening.


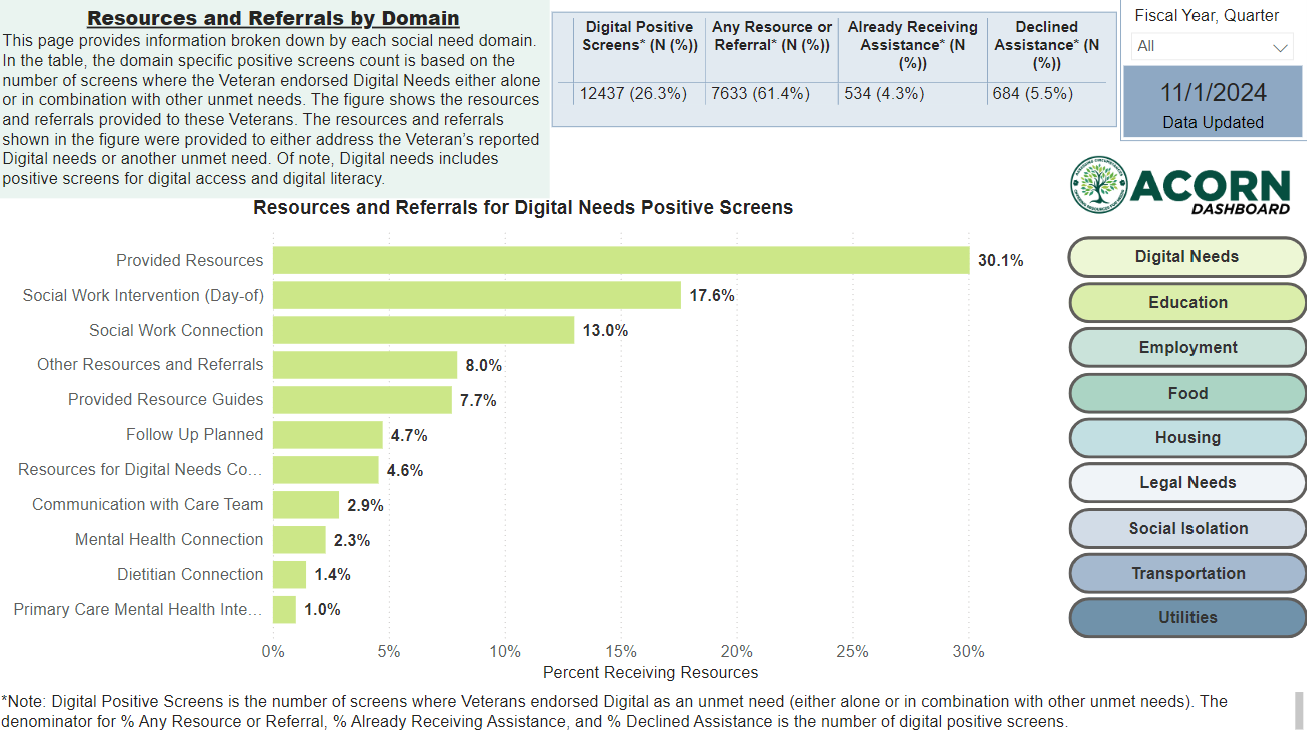


## 8. Patient-Level Data Page

The Patient-Level Data page displays total counts and percentages for unique living Veterans screened, positive screens, any resources or referrals provided, and screens not completed. Staff can view pertinent patient-level information for all Veterans who have completed at least one ACORN screen, including the date(s) of screening, endorsed social needs, the resources and referrals provided, Veteran demographic information, care team name(s), and appointment date(s).

For a full list of the table columns and data definitions, review the table in the Appendix.

#### **PHI Data Access**

Veteran PHI is only accessible on the Patient-Level Data page of the ACORN Dashboard, and staff will have access to patient information specific to their facility and in accordance with their data access permissions. Limited PII/PHI access is secured through Locally Secured Views (LSV), a CDW SQL database. Additional information on Dynamic LSV is available on the PowerBI SharePoint *[internal to VHA staff only]*.

The Privacy Act and HIPAA Privacy Rule protect PHI from being shared with anyone who does not have a right to the information. Following VHA’s best data practices and to ensure a Veteran’s privacy, **do not** share Veteran PHI with any staff who do not have the appropriate data access permissions.

Staff without the appropriate data access permissions will not be able to view any Veteran PHI data in the table. If you cannot see any data on the Patient-Level Data page and believe your permissions may be incorrect, visit the VHA Data Portal *[internal to VHA staff only].*

*Reminder:* Any filters applied on the other Dashboard pages will not be automatically applied on the Patient-Level Data page.

## 9. The ACORN Map

The ACORN Map page shows the settings and locations where ACORN has been implemented thus far. Each color on the map represents a unique clinical setting; the clinical settings are defined using stop codes. If the screening tool is being used in more than one clinical setting per location, multiple colors will be shown in the location circle.


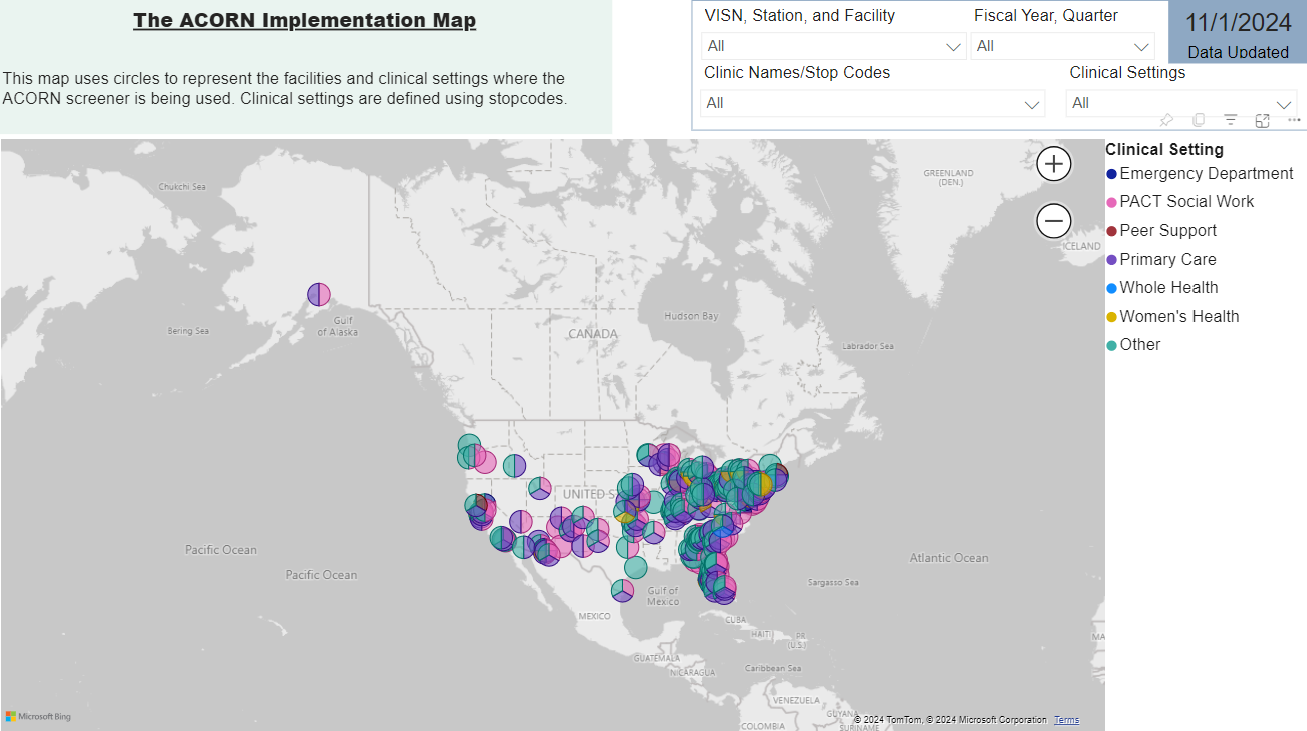


# **Frequency Asked Questions (FAQ)**

***How often does the ACORN Dashboard refresh?***

The Dashboard refreshes daily at 9:30am ET and 1:00pm ET. This means the most recent data are from the day prior. If the displayed data still have not refreshed by 2:00pm ET, contact the ACORN Dashboard Team at [VHAACORNDASHBOARD@va.gov](mailto:VHAACORNDASHBOARD@va.gov).

***Who can access the ACORN Dashboard?***

All VHA employees can access the Dashboard on the ACORN SharePoint *[internal to VHA staff only]* or directly through this link *[internal to VHA staff only]*.

***Why don’t I see my site listed in the dropdown list?***

For a site to appear on the dropdown list, at least one ACORN screen must have been completed at that facility. There is an approximately 24-hour lag between when the screen is completed in CPRS and when it appears in the ACORN Dashboard.

***Which filters are embedded in the Dashboard? How can I use the filters?***

There are several filters included in the Dashboard, including VISN, facility, and station as well as fiscal year and quarter and clinic name/stop code.

To select your VISN, Facility, and Station, use the down arrow to view the dropdown menu then click the relevant checkboxes for VISN, facility, and/or station. To select the Fiscal Year and Quarter, use the down arrow to view the dropdown menu then click the relevant checkboxes.

To select a clinic name and/or stop code, use the down arrow to view the dropdown menu then click the relevant checkboxes. Clicking on the “Clinic Names/Stop Codes” filter will also populate a search function at the top of the dropdown list that staff can use to search types of care or specific stop codes. Given the volume or clinic names and stop codes in VA, the filter is based on stop code classes.

*Note:* the filters on the Patient-Level Data page are not synced with the rest of the Dashboard. Any filters applied on the other Dashboard pages will not be automatically applied on the Patient-Level Data page.

***What is the difference between the “Unique Veterans Screens” table and the “Total Screens” table on the Overview Data page?***

The “Unique Veterans Screens” table only counts the number of unique Veterans who have been screened, and the “Total Screens” table shows the number of times the ACORN screening tool was completed. Since Veterans can be screened more than once, the count of Screens Done in “Total Screens” table will always be larger than the count of Unique Screens Done in the “Unique Screens” table.

***What does it mean if there is a row labeled ‘missing’ in a table?***

The data that create these tables are pulled directly from the Corporate Data Warehouse (CDW). If a Veteran’s information is missing in that database, then it will also appear as missing or null on the Dashboard. This is true across all pages on the Dashboard.

***How can I export pages of the Dashboard?***

The ribbon of at the top of the Dashboard has icons on the left and on the right. Clicking on the second option from the left, ‘Export,’ will open a dropdown. You can choose to export the pages in PowerPoint or PDF (the ‘Analyze in Excel’ feature is not enabled in the ACORN Dashboard). A pop-up will then appear with options to export current values or default values, as well as the option to export all pages or the current page.

***How can I export pages for a specific VISN, Facility, Station, and/or Fiscal Year and Quarter of
the Dashboard?***

To export and download pages from the Dashboard, click on the ‘Export’ option in the ribbon at the top of the Dashboard. Selecting ‘Current Values’ from the dropdown menu in that export pop-up window will download pages with the filters currently applied to the Dashboard.

***How can I export all the Demographics and/or Domain pages from the Dashboard?***

To export and download pages from the Dashboard, click on the ‘Export’ option in the ribbon at the top of the Dashboard. Unchecking ‘Exclude hidden report tabs’ in that export pop-up will download all the pages.

***Why does the ACORN Screens column show numbers fewer than 13 when the other columns
do not?***

ACORN Screens is an umbrella category and only denotes that a screening was completed, so it will display numbers fewer than 13 (see screenshot below). To protect Veteran privacy, Positive ACORN Screens, Any Resource or Referral, Already Receiving Assistance, and Declined Assistance counts are hidden in the ACORN Dashboard where totals are fewer than 13 (“<13” will appear in place of a value).


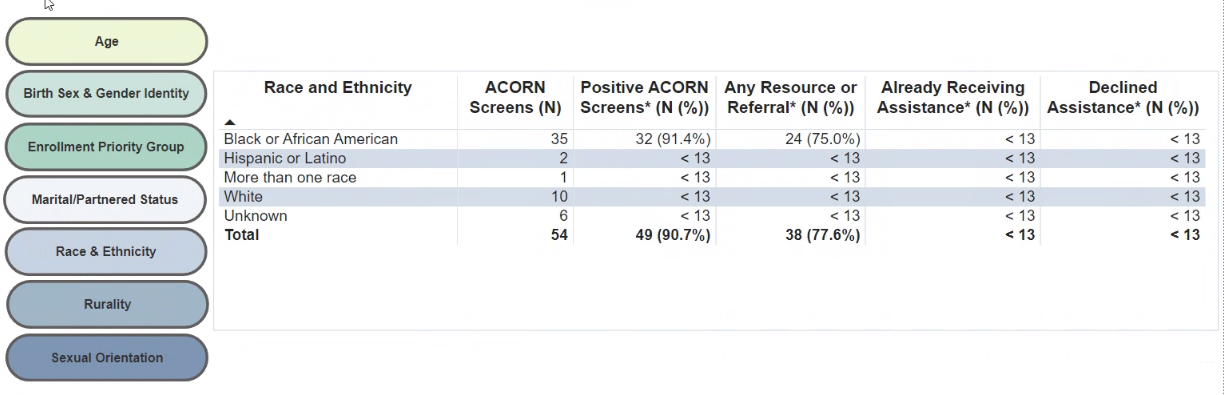


***A group is missing from at least one of the Demographic Characteristics tables, what does
this mean?***

If one category within a Demographic Characteristic table is missing from the rows, it indicates there are no data points for that category. For example, if a site has not screened any Veterans younger than 35, the age group 18-34 will not appear in the table.

***What does ‘Enrollment Priority Group’ mean?***

Enrollment Priority Groups are assigned by VA during Veteran enrollment. To learn more about Enrollment Priority Groups, visit the [VA Priority Groups webpage](https://www.va.gov/health-care/eligibility/priority-groups/).

***Why does the number of positive screens in the ACORN Dashboard differ from other specific health-related social needs VHA dashboards (like food security or homelessness)?***

The ACORN Dashboard only includes data from the clinical settings and sites using the ACORN screening tool. As a result, data in the ACORN Dashboard may differ from other VHA dashboards and is not considered representative of the full VHA-enrolled Veteran population.

ACORN is implemented in a range of outpatient primary care and specialty clinics, including PACT Social Work, Homeless PACT, Women’s Health, Emergency Departments, whereas the VHA clinical reminders for housing instability and food insecurity are administered annually to all VHA-enrolled Veterans. Thus, the data in the ACORN Dashboard includes a subset of VHA-enrolled Veterans who may or may not be at higher risk for certain health-related social needs.

***Can I access patient health information (PHI) in the Dashboard?***

Yes. The Patient-Level Data page is available for end users to view patient-level information. For additional information on the data available on the Patient-Level Data page, visit the Patient-Level Data Page section.

PHI is only accessible on the Patient-Level Data page of the ACORN Dashboard. All other pages of the Dashboard will display aggregate data at the national level.

***Which Veterans are included on the Patient-Level Data page?***

The Patient-Level Data page displays information only for living Veterans. Veterans who are deceased at the time of the most recent Dashboard update will not appear in the Patient-Level Data page table.

*Note*: Deceased Veterans are included in the data on all other Dashboard pages.

***Can I search for a specific Veteran by name on the Patient-Level Data page?***

Yes. Staff can search for specific patient information by using one of the four categories available in the Patient-Level Data Table Search box. The four categories are Veteran Name, PACT Name, Staff Name, and Screen Location (primary stop code class).

To search for a specific Veteran, enter their name into the search box, beginning with their last name. Veterans’ names are formatted on the Dashboard as Last Name, First Name, Middle Initial (if applicable).

***I opened the Patient-Level Data page and I can only see information from my site. Why can’t I see information about all Veterans who have been screened with ACORN?***

Following VHA’s best data practices, access to Veteran PHI is limited. Staff will only have access to patient information specific to their facility and in accordance with their data access permissions. Limited PII/PHI access is secured through Locally Secured Views (LSV), a CDW SQL database. Additional information on Dynamic LSV is available on the PowerBI SharePoint *[internal to VHA staff only]*.

The Privacy Act and HIPAA Privacy Rule protect PHI from being shared with anyone who does not have a right to the information. To ensure a Veteran’s privacy, **do not** share Veteran PHI with any staff who do not have the appropriate data access permissions.

***I opened the Patient-Level Data page and it is blank. Why can’t I see any data?***

Following VHA’s best data practices, access to Veteran PHI is limited. Staff will only have access to patient information specific to their facility and in accordance with their data access permissions. Staff without the appropriate data access permissions will not be able to view any Veteran PHI data in the table. If you cannot see any data on the Patient-Level Data page and believe your permissions may be incorrect, visit the VHA Data Portal *[internal to VHA staff only].*

***On the ACORN Implementation Map, are the circles representative of the number of ACORN screenings that have occurred in each clinical setting?***

The portion of each circle does not correlate to how many screens have been administered in that specific clinical setting.

***Who should I reach out to with questions about the Dashboard?***

For questions, concerns, or troubleshooting support about the ACORN Dashboard, contact the ACORN Dashboard Team at [VHAACORNDASHBOARD@va.gov](mailto:VHAACORNDASHBOARD@va.gov).

***Who should I reach out to with questions about ACORN that do not pertain to the Dashboard?***

If you have any questions about the ACORN initiative, contact the ACORN Leadership Team at [VHAACORN@va.gov](mailto:VHAACORN@va.gov).

# **ACORN Dashboard Data Definitions Table**

| Variable/Column Title | Brief Description | Additional Information (if applicable) | Data Source |
| --- | --- | --- | --- |
| Veteran Name | The Veteran's first and last name and middle initial. |  | Corporate Data Warehouse |
| Last 4 | The last four digits of the Veteran's social security number. |  | Corporate Data Warehouse |
| Phone Number | This is the most recent 10-digit phone number we have on record for the Veteran. This number is associated with the Veteran, not with a caregiver or family member. |  | Corporate Data Warehouse |
| Date of Most Recent Screen | Most recent ACORN screen date in Veteran's electronic health record. The date will be displayed as mm/dd/yy. |  | Corporate Data Warehouse |
| Date of Next Recommended Screen | This date is calculated based off the recommendation that the ACORN screener be administered on an annual basis and is calculated as one year from the date of the most recent screen. The date will be displayed as mm/dd/yy. |  | Corporate Data Warehouse |
| Date(s) of All Past Screens | Dates of any ACORN screens administered since July 1, 2021. The date(s) will be displayed as mm/dd/yy. |  | Corporate Data Warehouse |
| Positive Screen Domain(s) | This column shows the domain(s) for which a Veteran screened positive. If a Veteran screened positive for more than one domain, they will all be listed here. | Asterisk (*) means the data is pulled from a Veteran’s most recent ACORN screen. | Corporate Data Warehouse |
| Positive Acute Screen Domain(s) | This column shows the acute domain(s) for which a Veteran screened positive. If a Veteran screened positive for more than one acute domain, they will all be listed here. | Asterisk (*) means the data is pulled from a Veteran’s most recent ACORN screen. | Corporate Data Warehouse |
| Resources and Referrals Provided | This column shows all action steps associated with a Veteran's ACORN screen (provided resources, social work connection, provided resource guides, resources for digital needs connection, follow-up planned, social work intervention (day-of), mental health connection, communication with care team, dietitian connection, primary care mental health integration connection, other resources and referrals). | Asterisk (*) means the data is pulled from a Veteran’s most recent ACORN screen. | Corporate Data Warehouse |
| No Resources or Referrals Given | This column shows if a Veteran indicated no needs, declined assistance, and/or was already receiving assistance. | Asterisk (*) means the data is pulled from a Veteran’s most recent ACORN screen. | Corporate Data Warehouse |
| Staff Who Created the Screen | The name of the staff member who created the ACORN screening associated with a Veteran’s electronic medical record (e.g., CPRS). | Asterisk (*) means the data is pulled from a Veteran’s most recent ACORN screen. | Corporate Data Warehouse |
| Screen Location | This is the physical location associated with the visit during which the Veteran was screened. | Asterisk (*) means the data is pulled from a Veteran’s most recent ACORN screen. | Corporate Data Warehouse |
| Current Age | The numerical value in this field is calculated based on the Veteran's date of birth and reflects the Veteran's age on the date the data were last updated. It does not reflect the Veteran's age at time of their most recent ACORN screen. |  | Corporate Data Warehouse |
| Active Suicide Flag | This column indicates whether a Veteran has an active suicide flag. (Y/N) |  | Corporate Data Warehouse |
| CAN Score | The CAN score on this page is the CAN 2.5. We use the CAN 2.5 combined version of the 90-day hospitalization model and the 90-day mortality model. It is expressed as a percentile, ranging from 0 (lowest risk) to 99 (highest risk). Additional information regarding CAN can be found on the Clinical Assessment, Reporting and Tracking (CART) Program SharePoint site (internal only). | <https://dvagov.sharepoint.com/sites/VHACSDE/can> | Corporate Data Warehouse |
| Service Connection (%) | Service connection describes an injury or disease resulting in a disability linked to service in the military. A Veteran must also have separated or been discharged from service under other than dishonorable conditions. VA may grant service connection when evidence shows a link between a claimed medical condition and military service. It receives a rating of 0%, 10%, 20%, 30%, 40%, 50%, 60%, 70%, 80%, 90% or 100%. Additional information about service-connected disabilities can be found on VA's Disability Benefits website. | <https://www.va.gov/disability/about-disability-ratings/> | Corporate Data Warehouse |
| Sex | This field is populated based on the birth sex in the Veteran's electronic health record. |  | Corporate Data Warehouse |
| Combined Race/Ethnicity | Combined race and ethnicity is based on the racial and ethnic groups approved by the Office of Management and Budget (OMB) and currently used in VA. Race and ethnicity have been combined in the ACORN Dashboard per the Office of Health Equity’s approach to race and ethnicity categorization. This approach means that any Veteran who identifies as Hispanic or Latino appears only in that category and not in a race category, even if a race was selected in addition to Hispanic and Latino ethnicity in the Veteran’s medical record. |  | Corporate Data Warehouse |
| Rurality | Rurality refers to the Veteran’s rurality based on the address listed in their medical record. These data are based on the Rural-Urban Commuting Areas (RUCA) System developed by the U.S. Department of Agriculture and the Department of Health and Human Services. There are three categories: urban, rural, and highly rural. |  | Corporate Data Warehouse |
| Patient Aligned Care Team (PACT) Name | Patient Aligned Care Team (PACT) names come from the Reengineered Primary Care Management Modules in the CDW. Names were included if 'primary care' was mentioned in the Team Care Type column and if team roles were 'WH Primary Care Provider' or 'Primary Care Provider.' RPCMMTeamCareType like '%PRIMARY CARE%' and PrimaryProviderPCMSTDTeamRole_ID in (11,43) |  | Corporate Data Warehouse |
| Behavioral Health (BH) Team | Behavioral Health (BH) teams come from the Reengineered Primary Care Management Modules in the CDW. Teams were included if 'mh' or 'bhip' was mentioned in the RPCMMTeam column and if team roles were 'mental health,' 'primary care,' or 'primary care - hbpc.'  RPCMMTeamCareTypeCode in ('4','7','13') and (RPCMMTeam like '%mh %' or RPCMMTeam like '%mhtc%' or RPCMMTeam like '%bhip%') |  | Corporate Data Warehouse |
| Inpatient Specialty | Specialty contains information that describes the type of care an inpatient is receiving. It uses the name of the bed section, the name of the medical service, and the name of the treating specialty. | <https://vaww.virec.research.va.gov/CDW/Factbook/FB-CDW-Inpatient-Domain.pdf>  Asterisk (*) means the data is pulled from a Veteran’s most recent ACORN screen. | Corporate Data Warehouse |
| Last Primary Care (PC) Clinic | This is the name of the most recent primary care (PC) clinic the Veteran visited. Based on the following stop code combinations from the PACT Compass in VSSC:  Primary: 322, 323, 350; Secondary: any stop code  Primary: 103, 147, 148, 169, 178, 181, 182, 199, 216, 221, 229, 324, 325, 326, 424, 425, 428, 527, 528, 530, 536, 537, 542, 545, 546, 579, 584, 597, 611, 686; Secondary: 322, 323, 350  *322, 323, and 350 MUST be in either primary or secondary position |  | Corporate Data Warehouse |
| Last Primary Care (PC) Clinic Date | This is the date of the Veteran's most recent primary care (PC) clinic visit. |  | Corporate Data Warehouse |
| Next Primary Care (PC) Clinic | If a Veteran has a scheduled primary care (PC) clinic visit, the name will appear here. Based on the following stop code combinations from the PACT Compass in VSSC:  Primary: 322, 323, 350; Secondary: any stop code  Primary: 103, 147, 148, 169, 178, 181, 182, 199, 216, 221, 229, 324, 325, 326, 424, 425, 428, 527, 528, 530, 536, 537, 542, 545, 546, 579, 584, 597, 611, 686; Secondary: 322, 323, 350  *322, 323, and 350 MUST be in either primary or secondary position |  | Corporate Data Warehouse |
| Next Primary Care (PC) Clinic Date | If a Veteran has a scheduled primary care (PC) clinic visit, the visit date will appear here. |  | Corporate Data Warehouse |
| Last Social Work (SW) Clinic | This is the name of the most recent social work (SW) clinic the Veteran visited. Based on the following stop codes: Primary: 125 or 173 Credit: 125 |  | Corporate Data Warehouse |
| Last Social Work (SW) Clinic Date | This is the date of the Veteran's most recent social work (SW) clinic visit. |  | Corporate Data Warehouse |
| Next Social Work (SW) Clinic | If a Veteran has a scheduled social work (SW) clinic visit, the name will appear here. Based on the following stop codes: Primary: 125 or 173 Credit: 125 |  | Corporate Data Warehouse |
| Next Social Work (SW) Clinic Date | If a Veteran has a scheduled social work (SW) clinic visit, the visit date will appear here. |  | Corporate Data Warehouse |
| Last Mental Health (MH) Clinic | This is the name of the most recent mental health (MH) clinic the Veteran visited. Based on the following stop codes (located in either the primary or second location): Home Based Primary Care (HPBC): 156 or 157 General Mental Health: 502, 509, 510, 550, 557, or 558 Post Traumatic Stress Disorder (PTSD): 516, 519, 525, 540, 542, 561, 562, 580, or 581 Substance Use Disorder: 513, 514, 523, 545, 547, 548, or 560 Therapeutic and Supported Employment Services: 535, 536, 568, 573, 574, or 575 Psychosocial Rehabilitation and Recovery Centers: 582, 583, or 584 Mental Health Intensive Case Management: 546, 552, or 567 Primary Care Mental Health Integration: 534 or 539 Residential Care: 586, 587, 588, 593, 594, 595, 596, 597, 598, or 599 Other Mental Health: 292, 503, 505, 506, 512, 524, 527, 531, 532, 533, 537, 538, 553, 554, 559, 563, 564, 565, 566, 571, 572, 576, 577, 578, 579, 589, or 713 |  | Corporate Data Warehouse |
| Last Mental Health (MH) Clinic Date | This is the date of the Veteran's most recent mental health (MH) clinic visit. |  | Corporate Data Warehouse |
| Next Mental Health (MH) Clinic | If a Veteran has a scheduled mental health (MH) clinic visit, the name will appear here. Based on the following stop codes (located in either the primary or second location): Home Based Primary Care (HPBC): 156 or 157 General Mental Health: 502, 509, 510, 550, 557, or 558 Post Traumatic Stress Disorder (PTSD): 516, 519, 525, 540, 542, 561, 562, 580, or 581 Substance Use Disorder: 513, 514, 523, 545, 547, 548, or 560 Therapeutic and Supported Employment Services: 535, 536, 568, 573, 574, or 575 Psychosocial Rehabilitation and Recovery Centers: 582, 583, or 584 Mental Health Intensive Case Management: 546, 552, or 567 Primary Care Mental Health Integration: 534 or 539 Residential Care: 586, 587, 588, 593, 594, 595, 596, 597, 598, or 599 Other Mental Health: 292, 503, 505, 506, 512, 524, 527, 531, 532, 533, 537, 538, 553, 554, 559, 563, 564, 565, 566, 571, 572, 576, 577, 578, 579, 589, or 713 |  | Corporate Data Warehouse |
| Next Mental Health (MH) Clinic Date | If a Veteran has a scheduled mental health (MH) clinic visit, the visit date will appear here. |  | Corporate Data Warehouse |
| Last Nutrition Clinic | This is the name of the most recent nutrition clinic the Veteran visited. Based on the following stop codes:  Nutrition/Dietetics - Individual: 123 (primary or secondary) OR  Home Based Primary Care - Dietitian: 175 (primary) OR Weight Management and Move! Program - Individual: 372 (primary or secondary) |  | Corporate Data Warehouse |
| Last Nutrition Clinic Date | This is the date of the Veteran's most recent nutrition clinic visit. |  | Corporate Data Warehouse |
| Next Nutrition Clinic | This is the name of the most recent nutrition clinic the Veteran visited. Based on the following stop codes:  Nutrition/Dietetics - Individual: 123 (primary or secondary) OR  Home Based Primary Care - Dietitian: 175 (primary) OR Weight Management and Move! Program - Individual: 372 (primary or secondary) |  | Corporate Data Warehouse |
| Next Nutrition Clinic Date | If a Veteran has a scheduled nutrition clinic visit, the visit date will appear here. |  | Corporate Data Warehouse |
| Last Peer Support Clinic | This is the name of the most recent peer support (PS) clinic the Veteran visited. Based on the following stop code in the secondary location: 183 |  | Corporate Data Warehouse |
| Last Peer Support Clinic Date | This is the date of the Veteran's most recent peer support (PS) clinic visit. |  | Corporate Data Warehouse |
| Next Peer Support Clinic | If a Veteran has a scheduled peer support (PS) clinic visit, the name will appear here. Based on the following stop code in the secondary location: 183 |  | Corporate Data Warehouse |
| Next Peer Support Clinic Date | If a Veteran has a scheduled peer support (PS) clinic visit, the visit date will appear here. |  | Corporate Data Warehouse |
